# The audience shapes the information content of the honey bee waggle dance

**DOI:** 10.1073/pnas.2518687123

**Published:** 2026-03-23

**Authors:** Tao Lin, Shihao Dong, Gaoying Gu, Fu Zhang, Xiuchuan Ye, Tianyi Wang, Ziqi Wang, Jianjun Li, James C. Nieh, Lars Chittka, Ken Tan

**Affiliations:** ^a^Laboratory of Tropical Forest Ecology, Xishuangbanna Tropical Botanical Garden, Chinese Academy of Sciences, Mengla, Yunnan 666303, China; ^b^University of Chinese Academy of Sciences, Beijing 100049, China; ^c^School of Biological Sciences, Department of Ecology, Behavior, and Evolution, University of California, San Diego, CA 92093; ^d^Biological and Experimental Psychology, School of Biological and Behavioural Sciences, Queen Mary University of London, London E1 4NS, United Kingdom

**Keywords:** animal cognition, referential communication, signal encoding, social communication, spectator influence

## Abstract

The honey bee waggle dance, which encodes food location, is often thought to involve only one-way information transfer from dancers to signal receivers. Here, we show that the information content of the dance is influenced by the presence of followers and the number of appropriately aged potential followers in the hive, rather than the total number of bees on the dance floor. Dancers reduced the precision of directional and distance communication when they had fewer actual and potential followers. Even when the dance floor was crowded with young bees that do not follow dances, dancers showed the same declines in precision. These declines appear to arise as a byproduct of audience seeking during the return run, when dancers with fewer followers spent more time moving and covered greater distances across the dance floor. Although their mean waggle run duration and the distance covered per waggle run remained unchanged, both measures became markedly more variable when followers were scarce. Dancers likely use quorum sensing of tactile contacts and, potentially, age-specific odors to sense audience size. These results reveal that waggle dancing is a socially responsive process shaped by feedback from followers, demonstrating bidirectional information flow within this communication system.

## Significance

We show that the honey bee waggle dance changes depending on how many followers a dancer has and how many appropriately aged bees are available to follow it. When followers were scarce, dancers became less precise, even if the dance floor was crowded with young bees that do not follow dances. These declines in precision appear to arise because dancers search more widely for an audience, increasing their movement during the return run. The results suggest that dancers use simple social cues, such as tactile contacts, to sense follower availability. Thus, waggle dancing is not a one-way signal but a socially responsive behavior shaped by feedback from followers.

Animal communication is often framed from the sender’s perspective, yet receivers routinely shape signaling. However, audience effects, in which features of a signal change depending on the presence, composition, or engagement of potential receivers, are widespread across animals, including insects (1–5). For example, in the context of foraging, research by Miglietta et al. (6) on gorillas reveals that food calls, which are critical for indicating the availability of high-quality food sources and abundant food patches, are significantly influenced by the presence or absence of specific group members, such as the silverback and offspring. Drea and Carter (7) showed that the presence of conspecific spotted hyenas affected cooperation success, with experienced hyenas modifying their behavior to facilitate coordination with less experienced partners.

Honey bees (*Apis mellifera*) are an excellent invertebrate system for studying audience effects because of their sophisticated communication system, the waggle dance (8, 9). The waggle dance is a figure-eight motion in which the waggle run points in the direction of the resource relative to the sun’s azimuth (the direction to the sun projected onto the horizon). When a bee performs a vertical waggle run with its head pointing straight upward on the comb, it signals that the resource lies in the direction of the sun’s azimuth. Likewise, when the waggle run points 90° to the left of vertical, the resource is 90° to the left of the sun’s azimuth (8). Deviations in the waggle angle, therefore, decrease the precision of directional communication. In line with the path analogy, the duration of the waggle run increases as the distance to the food source increases, and greater variation in waggle run durations therefore communicates distance less precisely (8, 10).

Waggle dancers typically perform in a densely populated region of the colony called the “dance floor” close to the hive entrance, where potential dance followers congregate (8, 11). When a nectar forager returns to the nest, it begins seeking out bees to unload its food (12). Lindauer (13) observed that the likelihood and “vigor” of waggle dancing are influenced by the eagerness of nectar receivers to take a returning forager’s food load, and subsequent work showed that the behavior of nestmates, including nectar-transfer dynamics and trophallaxis (food exchange between nestmates), modulates the probability and strength of dancing, indicating that social feedback inside the hive already tunes whether and how strongly bees communicate (12, 14–16). However, beyond these audience effects, which alter the probability of a forager waggle dancing or change the number of dance circuits per dance bout, it was not known if the audience can alter the precision of the directional and distance information being communicated.

Shortly after the dance code was deciphered, von Frisch’s student Lindauer (13) observed that foragers struggling to find nectar receivers walked extensively across the comb, as if searching for food unloaders, and this difficulty in finding nectar receivers reduced their inclination to dance. However, given that signaling without signal receivers is futile, could dancers also be seeking followers? In *A. mellifera*, waggle dances occur in the darkness of the nest cavity, where followers must track the dancer’s movements by maintaining antennal contact with its abdomen (8). These tactile interactions provide the dancer with immediate feedback on how many bees are following. Dancers also sometimes pause to offer brief food samples to followers, and such trophallactic events could likewise signal the size of the follower group.

A complication is that the number of followers is dynamic and changes over time. Although bees may closely follow dancers for multiple successive runs to ensure accurate decoding of food location (17, 18), some followers depart after just one or two waggle runs (19). Thus, dancers cannot rely on a fixed number of followers throughout their performance and may need to recruit additional followers, particularly when the number of potential followers on the dance floor is low. Thus, how does their audience (followers) affect dance precision, and what do dancers do if they have an insufficient audience?

Here, we report on experiments testing if dance information content depends on the audience. We manipulated the size of the dance floor audience by removing (aspirating) nestmates to reduce the number of potential dance followers (experiment 1). To test if dancers were responding simply to the total number of bees on the dance floor, as opposed to the number of followers, we ran a different experiment (experiment 2) in which we maintained the total population size, but increased the number of very young bees that do not follow waggle dancers. Finally, we tested if the potential audience, defined as the number of appropriately aged bees that a dancer might expect to recruit on a given dance floor, could affect dancer behavior. In these experiments, we measured multiple parameters of the waggle dance, particularly the precision of communicating distance and direction. We also conducted four control experiments (control experiments 1-4) to test for potential disturbance effects of aspiration and two control analyses (control analyses 1 and 2) to test, respectively, the precision of our video tracking and to investigate the role of dancer orientation on dance imprecision.

## Results

### More Followers Increased Both the Likelihood of Waggle Dancing and the Number of Circuits Per Dance.

Does the number of followers affect the probability of a returning forager dancing and the number of dance circuits performed per dance? In experiment 1, we used an electric suction device (an aspirator) to remove and therefore manipulate the number of bees in the hive’s primary dancing area, the dance floor. We thereby created different audience size treatments: High_control_, Medium, Low, and High_recovery_ (see *SI Appendix*, Table S1 for detailed rationale). This setup allowed us to test for changes in forager behavior upon returning to the hive. Three independent control experiments found no evidence for significant aspiration disturbance effects. See *SI Appendix* for additional details on the observation hive setup (*SI Appendix*, Fig. S1), trophallaxis analyses (*SI Appendix*, Figs. S2 and S3), and aspiration disturbance control experiments (*SI Appendix*, Figs. S4–S6).

**Fig. 1.**
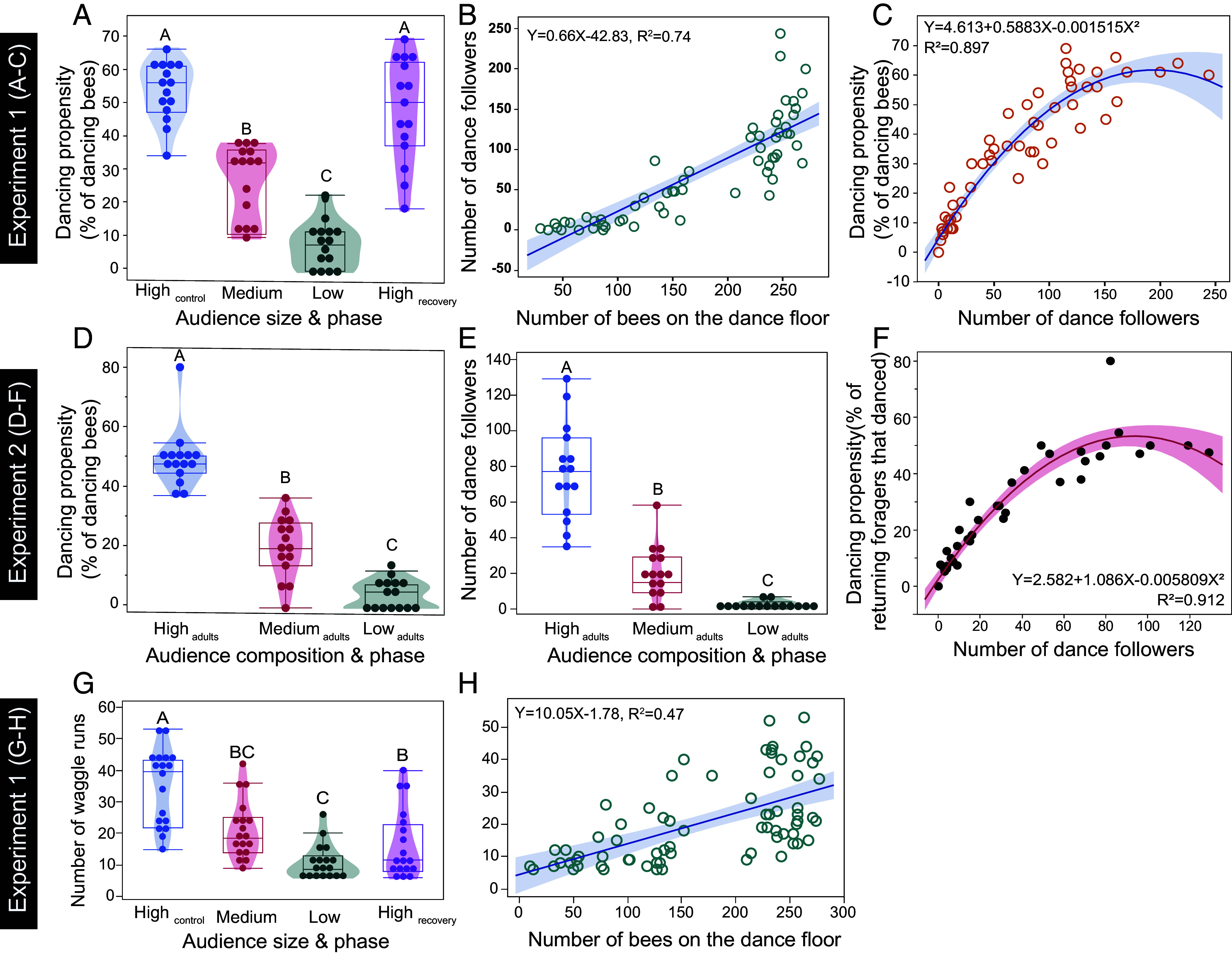
Increasing the audience size significantly increased waggle dancing. Experiment 1 (natural dance floor age composition) showed that (*A*) the propensity of returning foragers to dance increased when more bees were on the dance floor, and (*B*) the total number of followers on the dance floor was positively correlated with the number of bees on the dance floor. (*C*) Thus, the propensity to waggle dance was correlated with the total number of dance followers on the dance floor. The number of followers is important because in experiment 2 (young bee experiment), (*D*) dancing propensity decreased as the proportion of young bees (which do not follow dances) increased. (*E*) A key result is that even if the total number of bees on the dance floor was the same, the number of dance followers decreased when the proportion of young bees increased. (*F*) In experiment 2, dancing propensity also increased with the total number of dance followers on the dance floor. (*G*) In experiment 1, there were significantly fewer waggle runs in the Medium_phase_ and Low_phase_ as compared to the High_control phase_, and dancing did not fully recover in the High_recovery phase_. (*H*) In experiment 1, the number of waggle runs per dance is positively correlated with the number of followers per waggle run. All data points are shown (circles), and regression lines with 95% confidence intervals (shaded zones), box plots, and violin plots are shown. Different letters show significant differences [Tukey Honestly Significant Difference (HSD) test, *P* < 0.05].

A forager’s probability of dancing (dancing propensity) increases when it experiences greater demand for its collected nectar: when it waits less time for bees to unload its nectar (12) and when it receives more trophallactic contacts (20). In experiment 1, unloading delay time did not significantly vary with the audience size phase (*F*_3, 66_ = 1.30, *P* = 0.28, 8% colony effect, *R*^2^= 0.13), likely because our colonies were provisioned with enough stored food to remain healthy but were still motivated to collect additional resources. However, larger audience sizes increased the dancing propensity [*R*^2^ = 0.79, *F*_3, 54_ = 63.51, *P* < 0.0001^significant after Dunn-Sidak correction (DS)^, Fig. 1*A*]. Larger audience sizes also resulted in more followers (Fig. 1*B*), and thus we likewise saw a correlation between the propensity to waggle dance and the total number of followers on the dance floor (*F*_1, 56_ = 303.26, *P* < 0.0001, Fig. 1*C*).

Dancers also performed, on average, more waggle runs per performance when the initial audience size phase was larger than when it decreased (*F*_3, 23_ = 5.21, *P* = 0.007^DS^). There were no significant effects of unloading delay time (*F*_1, 1_ = 0.24, *P* = 0.70), or the number of trophallactic contacts (*F*_1, 56_ = 0.56, *P* = 0.46). No interactions were significant (*F*_3, 25_ ≤ 1.20, *P* ≥ 0.33, colony effect <1%, *R*^2^ = 0.59). Similarly, dancers performed more waggle runs per performance when they had more followers (*F*_1, 643_ = 37.98, *P*< 0.0001 ^DS^, Fig. 1*H*). There were no significant effects of unloading delay time (*F*_1, 28_ = 0.004, *P* = 0.95), or the number of trophallactic contacts (*F*_1, 64_ = 2.84, *P* = 0.10). No interactions were significant (*F*_1, 60_ ≤ 2.09, *P* ≥ 0.15, colony effect <1%, *R*^2^ = 0.54).

### Dancers Respond to Their Follower Count, Not to Total Bees On the Dance Floor.

But are dancers responding to their own followers or the total number of bees on the dance floor? Young bees (< 3 d of adult age) do not follow waggle dances (21). Thus, in experiment 2, we replaced different proportions of dance floor bees with such young bees while maintaining the same overall number of bees on the dance floor (no difference in total bee numbers per phase, *F*_2, 42_ = 1.32, *P* = 0.27, *SI Appendix*, Table S1). Dancing propensity significantly decreased as the number of young bees (which do not follow dances) increased on the dance floor (*F*_2, 40_ = 103.81, *P* < 0.0001^DS^, Fig. 1*D*). No young bees ever followed waggle dances, and the number of dance followers was negatively correlated with the proportion of young bees on the dance floor (*F*_1, 41_ = 112.43, *P* < 0.0001, colony effect <1%, *R*^2^ = 0.72).

However, in experiment 2, the audience size phase did not significantly alter the number of dance circuits: phase (*F*_1, 84_ = 0.70, *P* = 0.40), unloading delay time (*F*_1, 61_ = 2.06, *P* = 0.16), and the interaction unloading delay time × phase (*F*_1, 92_ = 0.01, *P* = 0.90) were not significant (colony effect <1%, *R*^2^ = 0.04). However, dancers performed more waggle runs per performance when there were more followers per waggle run (*F*_1, 29_ = 11.53, *P* = 0.002^DS^). Unloading delay time (*F*_1, 93_ < 0.00001, *P* = 0.998) and the interaction of unloading delay time × followers per waggle run (*F*_1, 4_ = 0.83, *P* = 0.41) were not significant (colony effect <1%, *R*^2^ = 0.07).

### Dancers Were More Accurate When They Had More Followers.

**Fig. 2.**
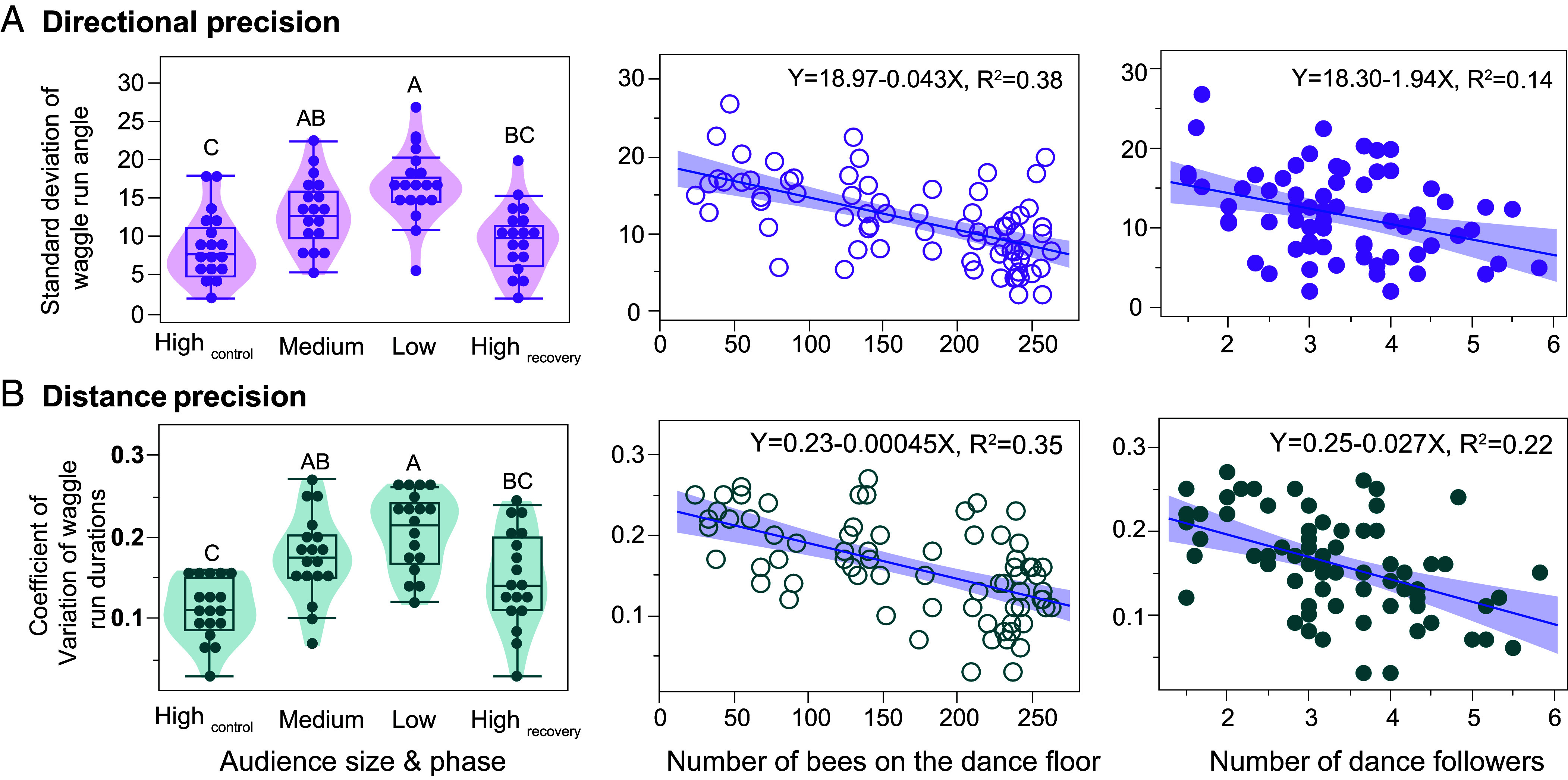
Larger audiences increased precision in waggle dance encoding of direction and distance (experiment 1). (*A*) Directional precision was quantified as the standard deviation of waggle-run angles (°), and (*B*) distance precision as the coefficient of variation of waggle-run durations. Directional and distance precision differed across audience-size phases (*Left* panels) and were correlated with both the number of bees on the dance floor (*Middle* panels) and the number of dance followers (*Right* panels). Different letters indicate significant differences (Tukey HSD test, *P* < 0.05).

In experiment 1, dancers indicated location more precisely when they had more dance followers (Movies S1 and S2). Directional precision increased when dancers had more followers because waggle angle standard deviations decreased (*F*_1, 66_ = 8.25, *P* = 0.006 ^DS^, Fig. 2*A*). There were no effects of the number of trophallactic contacts (*F*_1, 68_ = 1.15, *P* = 0.29), unloading delay time (*F*_1, 67_ = 0.13, *P* = 0.72), or any interactions (*F*_1, 63_ = 2.84, *P* ≥ 0.10, colony effect = 2%, *R*^2^ = 0.18).

Similarly, distance precision increased with the number of followers (waggle run duration coefficient of variation: *F*_1, 65_ = 17.50, *P* < 0.0001 ^DS^, Fig. 2*B*). The number of trophallactic contacts (*F*_1, 64_ = 2.45, *P*= 0.12) and unloading delay time (*F*_1, 55_ = 3.96, *P* = 0.05 ^not significant after DS correction^) were not significant. There was a significant interaction between the number of dance followers × unloading delay time (*F*_1, 65_ = 8.72, *P* = 0.004 ^DS^), but all other interaction terms were nonsignificant (*F*_1, 64_ ≤ 0.13, *P* ≥ 0.72, colony effect <1%, *R*^2^ = 0.35). The number of followers had greater explanatory power (log _worth_ = 4.06) than the interaction between the number of followers × unloading delay time (log _worth_ = 2.36). Detailed examination of the significant interaction showed that the positive effect of followers on distance precision was strongest when the unloading delay was short, weakened as the unloading delay increased, and disappeared above an unloading delay of approximately 26 s.

We replicated this experiment with a high-audience and low-audience dance floor on opposite sides of the same comb that were set up on the previous night before foragers returned to waggle dance (control experiment 4, see *SI Appendix*) and obtained the same results (*SI Appendix*, Fig. S7). In brief, waggle angle standard deviations were higher on the low-audience side (*F*_1, 35_ = 43.35, *P* < 0.0001 ^DS^), where dancers had fewer followers (*F*_1, 68_ = 134.91, *P* < 0.0001 ^DS^). Distance precision similarly decreased on the low-audience side: waggle run duration coefficient of variation (CV) was higher (*F*_1, 35_ = 15.61, *P* = 0.0004 ^DS^), and both the number of waggles per waggle run CV (*F*_1, 35_= 11.86, *P* = 0.0015 ^DS^) and the return run duration CV (*F*_1, 35_ = 12.07, *P* = 0.0014 ^DS^) were higher.

### Directional Precision Decreased As the Proportion of Very Young Bees Increased.

**Fig. 3.**
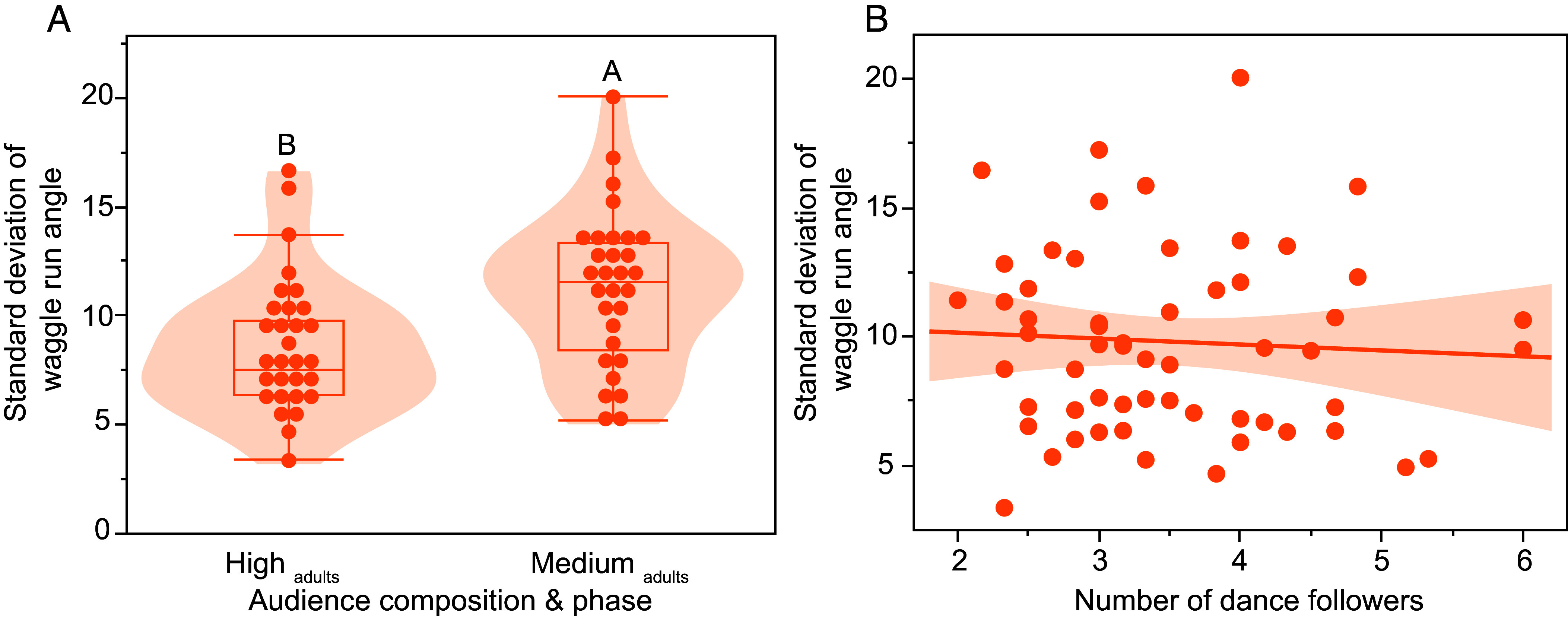
Decreasing the number of adult bees (which follow waggle dances) but maintaining the same total number of bees on the dance floor by increasing the proportion of young bees (which do not follow waggle dances) decreased dance precision (experiment 2). (*A*) An audience containing a high proportion of young bees (41% in the Medium_adults phase_), and consequently fewer adult bees capable of following dances, led to significantly greater directional error in waggle dances compared to an audience composed of 100% adults (High_adults phase_). (*B*) Waggle angle standard deviations were not affected by the number of dance followers. Different letters show significant differences (Tukey HSD test, *P* < 0.05).

In experiment 2, the number of dance followers significantly decreased as the proportion of young bees increased (see above), and we therefore observed that dances had lower directional precision (larger waggle angle standard deviations) in the Medium_adults phase_ than in the High_adults phase_ (*F*_1, 54_ = 10.06, *P* = 0.0026 ^DS^, Fig. 3*A* and Movies S3 and S4). Unloading delay time (*F*_1, 56_ = 1.14, *P* = 0.29), the total number of trophallactic contacts (*F*_1, 56_ = 0.005, *P* = 0.94), and all interaction terms (*F*_1, 52_ ≤ 2.07, *P* ≥ 0.16, colony effect <1%, *R*^2^ = 0.14) were not significant.

However, waggle angle standard deviations increased with the proportion of young bees on the dance floor (*F*_1, 54_ = 7.78, *P* = 0.007 ^DS^), but there were no significant effects of unloading delay time (*F*_1, 56_ = 1.17, *P* = 0.28), total trophallactic contacts (*F*_1, 56_ = 0.002, *P* = 0.97), or any interactions (*F*_1, 52_ ≤ 1.94, *P* ≥ 0.21, colony effect <1%, *R*^2^ = 0.11).

However, in experiment 2, the audience phase did not significantly affect distance precision (waggle run duration CV: *F*_1, 54_ = 0.58, *P* = 0.45). Unloading delay time (*F*_1, 54_ = 0.0006, *P* = 0.98), total trophallactic contacts (*F*_1, 52_ = 0.03, *P* = 0.86), and interactions (*F*_1, 52_ ≤ 1.23, *P* ≥ 0.37, colony effect <1%, *R*^2^ = 0.15) were also not significant.

We then looked at the effects of the number of followers. Directional precision was not affected by the number of followers (waggle angle standard deviations: *F*_1, 12_ = 0.39, *P* = 0.39, Fig. 3*B*), unloading delay time (*F*_1, 56_ = 0.55, *P* = 0.46), total trophallactic contacts (*F*_1, 53_ = 0.04, *P* = 0.85), or any interactions (*F*_1, 52_ ≤ 1.00, *P* ≥ 0.32, colony effect <1%, *R*^2^ = 0.08). Similarly, distance precision was not affected by the number of followers (*F*_1, 52_ = 1.13, *P* = 0.29), unloading delay time (*F*_1, 56_ = 0.02, *P*= 0.88), total trophallactic contacts (*F*_1, 54_ = 0.06, *P* = 0.82), or any of the interactions (*F*_1, 52_ ≤ 1.13, *P* ≥ 0.29, colony effect <1%, *R*^2^ = 0.03). For these analyses, we did not use the Low_adults phase_ because very few bees danced during this phase.

### Dancers Were Less Accurate When the Potential Audience Was Smaller.

Our data showed that dancers respond to the number of dance followers, but this raised an interesting question. There was a correlation between the number of bees on the dance floor and the number of dance followers, but it was not perfect (*R*^2^= 0.74), and there was high variation even at the highest number of 250 adult bees (Fig. 1*B*). Thus, even when there were many potential followers, some dancers attracted only a few and, conversely, when there were fewer potential followers, some dancers attracted several. Does the number of dance followers that a dancer might expect to attract, what we call the “potential audience”, affect dancers?

**Fig. 4.**
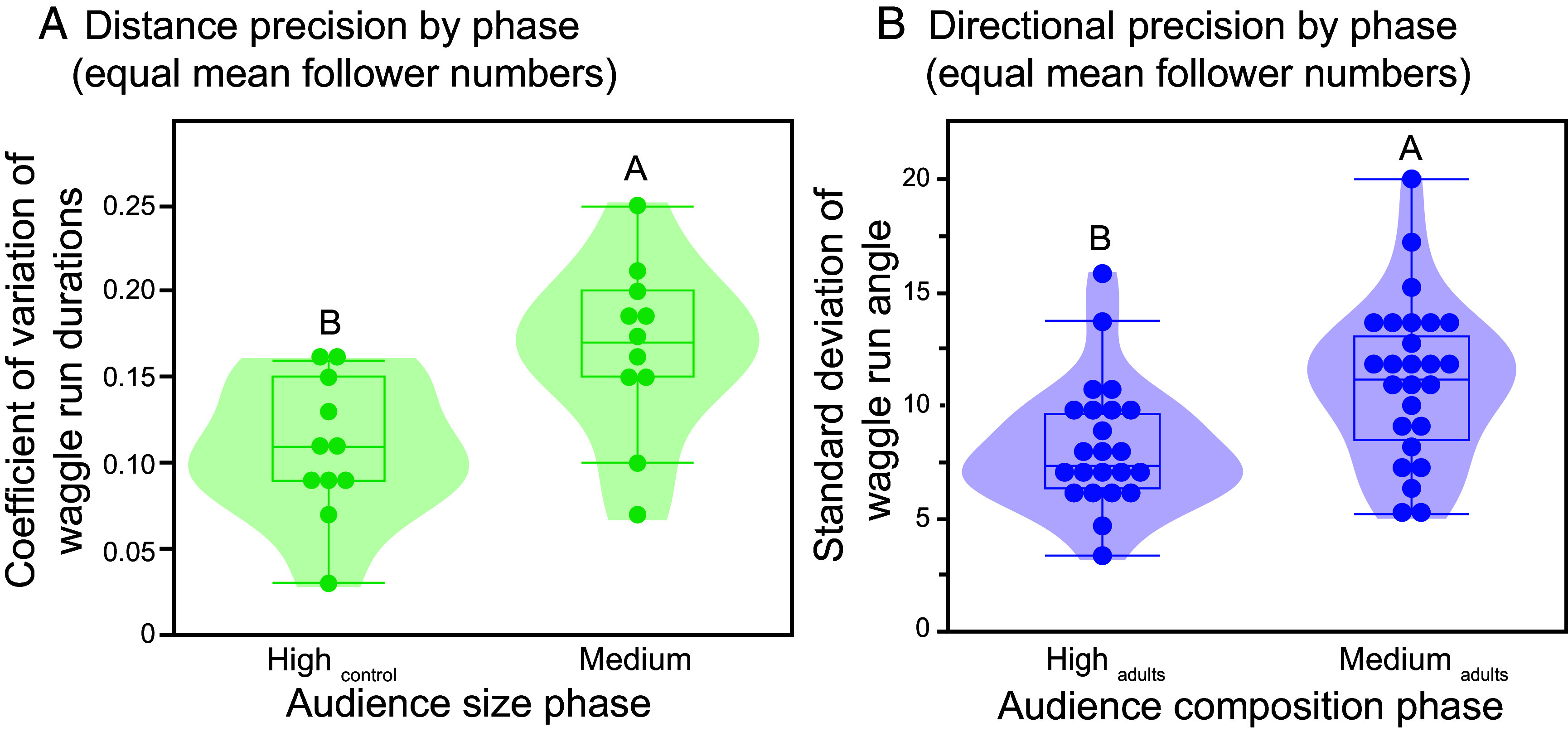
When dancers had the same average number of followers, but had greater difficulty in obtaining this audience because there were fewer adult bees on the dance floor (medium phase), distance and directional precision decreased. As the number of potential dance followers decreased (*A*) variation in waggle run duration increased (experiment 1) and (*B*) waggle angle standard deviations increased (experiment 2). Different letters indicate significant differences (Tukey HSD test, *P* < 0.05).

To address this question, we used our data from experiment 1 and compared waggle dances performed under conditions in which they had different numbers of adult bees on the dance floor but ended up with the same average number of dance followers (2 to 5 followers per dance). Bees in the High_control phase_ and Medium_phase_ had respectively 3.5 ± 0.7 followers per dance and 3.6 ± 0.7 followers per dance (not significantly different: *F*_1, 17_ = 0.03, *P* = 0.87, colony effect = 13%). In the Medium_phase_, even though dancers had the same average number of followers, both waggle run duration CV (*F*_1, 17_ = 9.03, *P* = 0.009 ^DS^, colony effect <1%, Fig. 4*A*) and return run duration CV (*F*_1, 19_ = 7.66, *P* = 0.01 ^DS^, colony effect <1%) increased as compared to the High_control phase_. However, waggle angle standard deviations did not change (*F*_1, 16_ = 1.79, *P* = 0.20, colony effect <1%).

We also looked at the data from experiment 2 using the same criteria. Bees in the High_adults phase_ and Medium_adults phase_ had respectively 3.3 ± 0.6 followers per dance and 3.3 ± 0.7 followers per dance (not significantly different: *F*_1, 46_ = 0.006, *P* = 0.93, colony effect <1%). In the Medium_adults phase_, although dancers had the same average number of followers, waggle angle standard deviations were significantly greater (*F*_1, 45_ = 9.22, *P* = 0.004 ^DS^, colony effect <1%, Fig. 4*B*) as compared to the High_adults phase_. Waggle run duration CV (*F*_1, 45_ = 1.28, *P* = 0.26, colony effect <1%) and return run duration CV (*F*_1, 46_ = 0.02, *P* = 0.89, colony effect <1%) were not significantly different between phases. These results suggest that, beyond the number of individuals currently following the dance, the potential audience can influence the precision of waggle dancing.

#### Audience seeking behavior.

But why should the potential audience (bees not directly engaged with dancing bees) affect the precision of communication signals? When dancers encountered a low-potential audience, they seemed to exhibit “audience seeking” behavior: they tended to interrupt their waggle runs and move around more, covering a greater total distance on the dance floor and thereby encountering more bees than when they were on a high audience dance floor. We therefore measured these behaviors. For example, in experiment 1, when there were fewer bees on the dance floor, dancers increased variation in return run durations (higher coefficient of variation, *F*_1, 69_ = 23.59, *P* < 0.0001 ^DS^, colony effect <1%, *R*^2^ = 0.26) and in the variation of waggle run durations (*F*_1, 68_= 35.54, *P* < 0.0001 ^DS^, colony effect <1%, *R*^2^ = 0.34).

We also measured the distance covered and the duration of the period in which they produced their first six waggle runs when exposed to larger (High_control phase_) and smaller (Low_phase_) audiences in experiment 1. Dancers exposed to smaller audiences spent significantly longer in their return runs (*F*_1, 55_ = 35.75, *P* < 0.0001), had significantly greater variation in return run durations (return duration CV: *F*_1, 55_ = 48.46, *P* < 0.0001), covered greater distances in their return runs (*F*_1, 56_ = 52.81, *P* < 0.0001), and had greater variability in the return distances covered (*F*_1, 55_ = 54.03, *P* < 0.0001) than dancers exposed to larger audiences (Movies S5 and S6). In addition, the speed of the return run increased when there were more bees on the dance floor (audience phase effect: *F*_1, 55_ = 4.81, *P* = 0.03, colony effect = 10%, and *R*^2^ = 0.16).

**Fig. 5.**
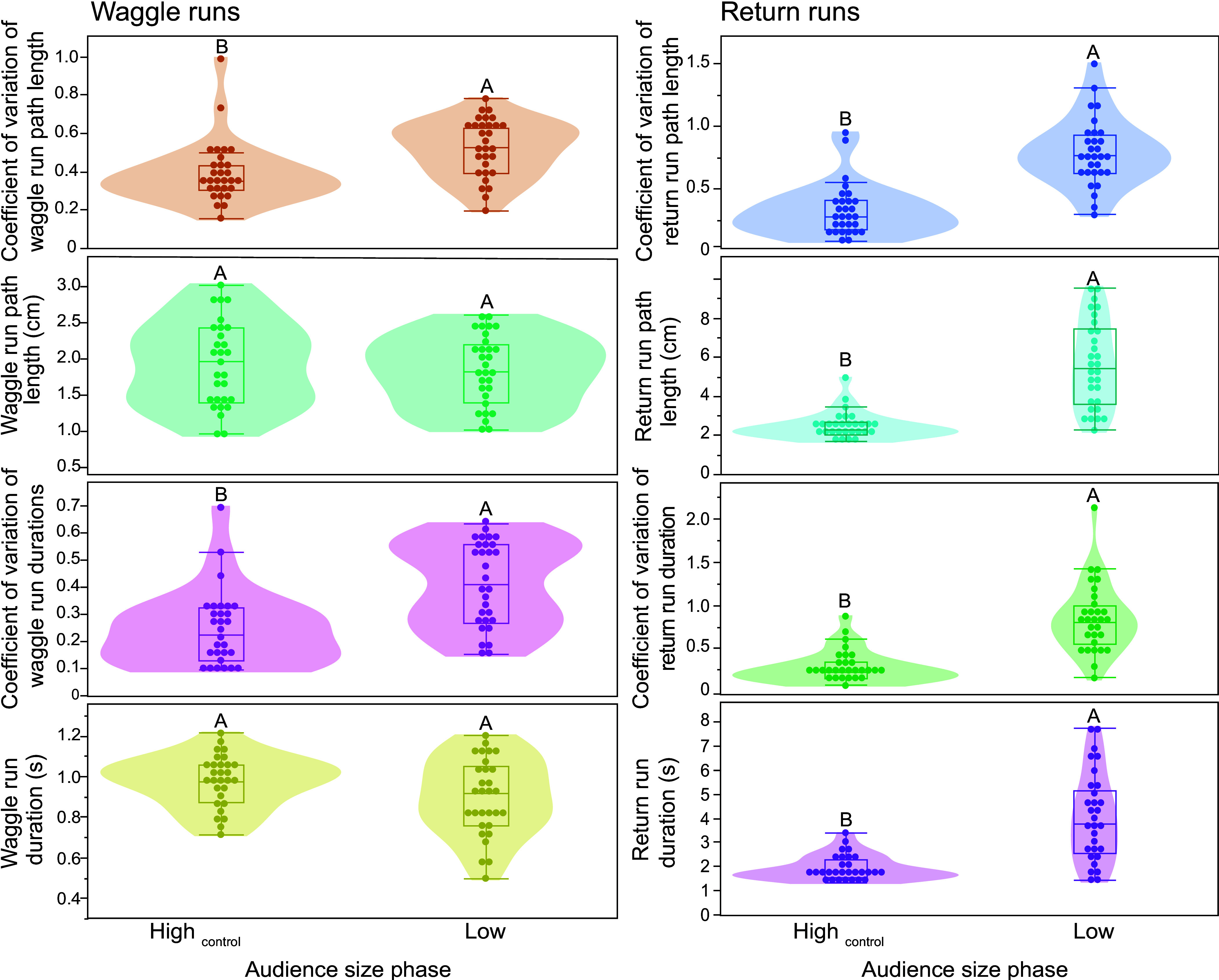
Audience seeking behavior altered waggle dancing because bees walked farther distances and therefore covered greater distances (Movies S5 and S6) during their dance return runs when the dance floor had fewer followers (experiment 1). Mean waggle duration did not differ across audience sizes, but both the variability of waggle durations and the variability of distances covered during waggle runs increased under low-audience conditions. In the low_phase_, dancers spent more time and traveled significantly farther during their return runs when follower numbers were low. Different letters indicate significant differences (Tukey HSD test, *P* < 0.05).

In these tests, colony effect ≤5%, and *R*^2^ ≥ 0.25. In contrast, the distance communicated did not significantly change (waggle duration mean: *F*_1, 55_ = 3.09, *P* = 0.08; waggle duration distance covered: *F*_1, 55_ = 0.63, *P* = 0.43; colony effect <1%, and *R*^2^ ≤ 0.003), although it was more variable (waggle duration CV: *F*_1, 55_ = 19.74, *P* < 0.0001; waggle duration distance covered CV: *F*_1, 55_ = 10.92, *P* = 0.002, colony effect ≤8%, and *R*^2^ ≥ 0.13, Fig. 5).

#### Larger numbers of bees on the dance floor resulted in more trophallaxis and longer contacts, but primarily with older workers.

Next, we tested the hypothesis that audience size will alter forager trophallaxis. Specifically, we sought to determine how dancing bees might assess the size of their potential audience and whether trophallactic contacts could provide information that allows them to distinguish young from older bees. In experiment 1, the number of total trophallactic contacts significantly increased with the number of bees on the dance floor (*F*_1, 67_ = 10.25, *P* = 0.002 ^DS^, *SI Appendix*, Fig. S2), although there were no differences between the number of such contacts between phases and no correlation between the number of trophallactic contacts and the number of followers (see above).

Trophallaxis duration was also not significantly influenced by audience size phase (*F*_3, 65_ = 1.45, *P* = 0.24, *SI Appendix*, Fig. S2). In experiment 2, upon returning to the hive, foragers primarily engaged in trophallaxis with adult bees and rarely with young bees (paired two-tailed *t* test, *t*_95_ = −22.44, *P* < 0.0001), and spent significantly more time per trophallactic contact with adults than with young bees (paired two-tailed *t* test, *t*_95_= −31.55, *P*< 0.0001, *SI Appendix*, Fig. S3).

## Discussion

Our experiments show that waggle dancers with fewer followers perform fewer dance circuits and encode direction and distance less precisely than dancers with more followers. Dancers responded specifically to the number of followers and to the availability of appropriately aged bees that could become followers, rather than to the total number of bees on the dance floor. When we held follower number constant but reduced the pool of potential followers by increasing the proportion of young bees that rarely follow dances, dancers again became less precise. Thus, both immediate audience size and the size of the potential audience modulate the precision of this referential signal. The differences we report arose specifically from variation in follower number and engagement, confirming that the audience effects observed reflect social interactions among bees.

These findings extend earlier work on social feedback and recruitment, which showed that unloading wait times and trophallaxis shape the probability of waggle dancing and the number of waggle runs produced per dance, but did not examine the precision of the spatial encoding (12, 14, 16). Although unloading delay time and the number of trophallactic contacts can explain the probability of a forager deciding to dance and the number of waggle runs it will produce (12, 14), they do not explain directional or distance error in the dance. We note that unloading wait times did not vary between phases in experiments 1 and 2, likely because there were still bees seeking the nectar being brought in, even at lower dance floor populations, likely because nectar receivers remained available and interested in the incoming nectar even when dance floor populations were lower. We established colonies with food stores sufficient for their health, but with enough need for additional nectar to allow bees to be trained readily to the feeders.

The one exception is that having more followers can increase distance precision when the unloading delay is short, but this increase in precision disappears above an unloading delay of approximately 26 s. Thus, our data support a strong effect of the number of followers on the precision of spatial encoding, but other factors clearly also affect this precision. For example, De Marco et al. (22) showed that fluctuating nectar rewards can transiently increase the waggle dance waggle angle standard deviation, an effect linked to changes in motivational state, which is also likely impacted by the unloading wait time, given that bees are less motivated to dance if they must wait longer to have their food unloaded (12).

In experiments 1 and 2, there were no significant correlations between the number of dance followers and the total number of trophallactic events that a forager received after returning to the nest with nectar until it departed again to collect more nectar. We counted the total number of trophallactic events because food unloading and trophallaxis before dancing affect the propensity to dance (12) and are correlated with the number of dance circuits performed. Farina and Wainselboim (23) showed that the dance followers engaged in shorter trophallactic contacts than non-followers, and only 26% of dance followers received food as compared to 58% of non-followers. Our results confirm that trophallaxis between followers and the dancers is not a major component of the total number of trophallactic events that a forager receives during its return to the nest. However, such trophallaxis, although limited, could provide the dancer with some information about how many followers it has.

But why are bee dances less precise when there are fewer followers? We interpret this reduced precision not as an adaptive adjustment of the message but as a constraint that arises when dancers must search for an audience while continuing to signal. When followers were scarce, dancers lengthened and expanded their return runs, moving over larger areas of the comb and repeatedly restarting waggle runs on new terrain. This audience seeking behavior likely increased the variability of waggle durations, distances, and waggle angle standard deviations. Given that horizontal waggle runs have greater angular scatter than vertical ones and because local comb irregularities are known to degrade dance precision (24–26), frequent relocation across the comb could make it harder for dancers to maintain identical posture and foothold across runs. In contrast, when audiences were abundant, return runs were shorter and more localized, allowing dancers to reuse the same patch of comb and maintain a more stable motor pattern. We therefore regard the increased directional and distance error at low follower numbers as a mechanical and informational cost of audience seeking, not as an evolved strategy to reduce accuracy.

This interpretation is also supported by the young bee experiment (experiment 2). In this experiment, the total density of bees on the dance floor (and therefore microclimatic conditions such as temperature and humidity that might affect dancing) remained similar across phases, but directional scatter increased when the proportion of young bees rose and follower numbers consequently declined. Unloading delay time did not differ significantly across audience treatments and did not predict dance precision. Instead, dancers behaved as if they were constrained by the difficulty of obtaining and retaining an appropriate audience, even though the comb was just as crowded. The movement of the dancers was likely not constrained by the density of bees on the dance floor because dancer return runs were significantly faster, by about 17% on more crowded dance floors. We considered whether dancers were simply searching for food unloaders rather than an audience of followers. However, in both experiment 1 and experiment 2, unloading wait time was not affected by audience size phase, indicating that returning foragers did not experience greater difficulty in finding nectar receivers when the audience was small. Moreover, unloading delay did not predict dance precision in our models, whereas follower number and potential follower availability did. Together, from these results, we argue against the idea that increased walking and reduced precision at low audience sizes are driven primarily by foragers seeking unloaders, and instead support the interpretation that these patterns arise from seeking followers.

Follower availability could, in principle, convey functionally relevant information. When few followers are available, the colony may already have many foragers engaged, so reduced precision could promote exploration of additional food locations. When many followers are available, the colony is more likely to have spare foraging capacity, so increased precision could recruit more efficiently and help exploit the advertised resource. However, our results are more consistent with the idea that reduced precision arises as a byproduct of the behavioral demands of acquiring and retaining receivers on the dance floor. If dancers were simply tuning scatter as an adaptive feature, they could remain in one place and increase variation in waggle run direction and duration. Instead, when followers are scarce, dancers show longer and more spatially extensive return runs, travel farther across the dance floor, and exhibit more interruptions and restarts, consistent with a cost of audience seeking.

Overall, our results support the constraints explanation of dance error: imperfections in the waggle dance occur when dancers must simultaneously maintain a spatial signal and search for followers. Audience seeking and precise signaling may draw on the same behavioral and motor resources, and under low audience conditions, the demands of searching for followers evidently compromise the dance precision.

How might dancers estimate audience size and composition? Waggle dancers could use quorum sensing of their social environment. Followers interact with dancers through stereotyped antennal contacts and body touches that encode directional information for the follower (27–29). The dancer can, in turn, assess the level of such contact during early waggle runs and, when contacts are sparse, end their waggle runs and embark on longer and more meandering return paths that search for additional followers. Within a dance bout, several cues could inform the dancer about how easy it is to obtain and retain followers. The number and rate of trophallactic contacts provide information about colony interest in the advertised nectar and about the availability of potential followers, even though our data show that trophallaxis itself does not drive the observed changes in precision (30, 31). During audience seeking, dancers traverse larger areas of the comb, encountering many bees whose characteristic orientation relative to the dancer may signal their follower status (32).

However, we also found that the dance precision was affected by the potential audience. Even when dancers had the same average number of followers, errors increased when they had a smaller potential audience. In experiment 1, waggle run and return run durations became significantly more variable when bees sensed fewer followers. In contrast, experiment 2 (the young bee experiment) showed that only the waggle angle standard deviation was more variable under smaller potential audiences. We suggest that in experiment 1, the low number of bees on the dance floor made it easier for dancers to estimate their prospective audience based on cues such as density and overall activity. By comparison, in experiment 2, although the number of bees remained constant, most were young bees with no inclination to follow dances. Dancers, therefore, had to distinguish “potential” from “nonpotential” followers.

But how could bees gauge their potential audience? Returning foragers could also gauge how crowded and active the dance floor is from antennal contacts with nestmates and from the ease of finding followers, in a manner analogous to quorum sensing in ants and in honey bee swarm nest site selection, where encounter rates and local density govern recruitment mode switches and lift off decisions (33–35). However, the young bee experiment (experiment 2) shows that tactile density cues alone may not be sufficient because overall bee density remained constant. Younger bees differ from older foragers in their cuticular hydrocarbon profiles, which are shaped by both intrinsic programs and colony environment and change as workers mature into foragers (36). Waggle dancers could therefore use age-linked hydrocarbon blends and associated volatiles, sampled through repeated antennal contacts and local odor fields, to distinguish potential followers from non-followers as they walk through the crowd.

Dance precision is further influenced by internal physiology and external conditions. Biogenic amines such as dopamine, for example, can modulate locomotor kinematics and may therefore affect how reliably a bee can repeat a waggle vector (37). De Marco et al. (22) showed that unstable food quality, in the form of fluctuating sucrose concentrations, increased waggle angle standard deviations relative to a constant high reward, again indicating that motivational state and environmental predictability can reduce precision. Our study adds social context to this list. Reduced precision emerged when audience size and composition limited the expected benefits of recruitment, in a pattern reminiscent of bees that initially increase directional scatter for less profitable resources before refining their dances.

Our findings on audience seeking behavior raise another question: what is the function of the waggle dance return run? Since all spatial information is conveyed during the waggle run, the turns at its start and end may help recruits gauge its duration. Yet a dancer could, in theory, briefly pause between waggle runs to mark start and stop points. Followers might track such a dancer more easily. In addition, De Marco et al. (22) showed that the angular divergence between successive waggle runs depends on the sequence of left and right turns, indicating that the reorientations between waggle runs contribute to directional scatter. The return run may therefore help the dancer remain in approximately the same region of comb, but this advantage likely comes at the cost of some loss of directional precision. Why, then, do dancers not simply proceed in a straight path, with brief pauses, until they reach the comb edge and then walk back? Dormagen et al. (38) recently showed that dancers become spatially segregated on the dance floor and suggested that such segregation may help followers locate dances advertising similar food sources. A waggle dance without a return run, in which dancers advance steadily across the comb, could in principle amplify this spatial sorting effect. We propose that the return run serves an additional and more important function: audience seeking. These pauses between spatial communication offer opportunities to attract new followers, which is essential for effective communication. Our results, therefore, highlight the importance of the audience for the precision of location communication and the tension between audience seeking and signaling.

## Materials and Methods

### Location and Colonies.

Bee colonies were housed at the bee apiary in the Southwest Center for Biological Diversity, Chinese Academy of Sciences (Kunming, China). We created each colony by transferring two combs (43.5 × 23 cm) and the queen from the source colony to create experimental colonies (*SI Appendix*, Fig. S1), each containing approximately 1,200 bees over 14 d of age, a small amount of nectar and pollen, and capped brood. We created these colonies with bees >14 d old so that we could fully control the number of young bees that were in each colony for experiment 2. We used a different source colony to create each observation colony, and all queens came from the same queen breeder. In addition, we maintained colonies with a similar genetic background (obtained from the same queen breeder) to supply age-marked bees for our observation colonies. Source colonies were maintained using standard methods (39), and all observation colonies were in good health and required no treatments. The colony entrance was placed in the bottom right area of the observation hive and was connected to the outside through a 2.2 cm inner diameter and 25 cm long tube through the wall. All colonies had approximately equal honey and pollen stores (based on visual inspections) and were healthy and queen-right. All colonies also had empty comb space in each frame, including in the dance floor, to store additional nectar and honey. For all colonies, it was relatively easy to train foragers to artificial feeders, and they usually returned to the nest after foraging and then waggle danced. Thus, the colonies had space to store nectar and honey and a demonstrated need for it. No supplemental food was provided to the colonies for the duration of the experiment. Sample sizes for these experiments and all control experiments and analyses are shown in *SI Appendix*, Table S2. The full dataset is available at Zenodo.org at https://doi.org/10.5281/zenodo.10892587.

We conducted two main experiments, usually three days after the colonies were set up (see sample sizes and colony replication in *SI Appendix*, Table S2). We conducted our trials for both experiments (April–September) on sunny days with moderate temperatures (20 to 26 °C). In experiment 1, we reduced the number of bees on the dance floor (April–June 2023). In experiment 2, we tested if waggle dancers respond to the presence of any bees on the dance floor or to bees that follow waggle dances (older bees): we therefore placed young bees that do not follow waggle dances on the dance floor (July–September 2023). To obtain young bees, combs with late-stage (purple-eyed pupae) were removed from healthy, queen-right colonies and placed inside a box in an incubator (PRX-250B, Ningbo Saifu Experimental Instrument Co., Ltd.) for 24 h (dark environment, 34 °C, and 75% relative humidity). As soon as they emerged, young bees were marked with paint on their thoraces and transferred to a two-frame observation hive. In this experiment, to ensure that no younger bees were present, we removed and replaced all frames containing open or capped brood twice weekly for two wk before cohort introduction and continued to exclude brood-containing frames during the trial. Only color-marked cohort workers were sampled; unmarked or callow bees were excluded.

In our preliminary experiments, we found that newly emerged young bees introduced from a different colony could sometimes be removed by older bees from the observation colony and that the young bees could sometimes try to spend time inside comb cells, perhaps to escape being removed by other bees. However, this behavior limited our ability to determine their age based on seeing their paint marks. Therefore, we placed the young bees in the observation hive approximately 14 to 15 h in advance of the actual trial. After this period of time, the number of young bees stabilized and no longer spent much time inside cells, so we could more accurately measure their numbers.

### Experiment 1: Audience Effects On the Honey Bee Waggle Dance.

Between 10:00 AM and 12:00 PM on each experimental day, we trained about 30 foraging bees from the focal observation colony to a feeder providing 50% (w/v) sugar solution and placed 500 m from the colony. The bee training method described by Dong et al. (40) is highly effective for training forager bees to a distant feeder rapidly. This method is more efficient than gradually training bees away from their colony by moving the feeder incrementally. During training, forager bees were captured at the observation hive entrance using an insect net and transported to a feeding site. The feeder, identical to that described by Dong et al. (40), consisted of an inverted 70 mL sugar-water reservoir vial and a plastic grooved plate with a 2 cm wide water trough at the base, circular in shape with an 8 cm diameter, accommodating approximately 20 bees simultaneously. Bees were transferred from the insect net to the feeder via a 10 mL centrifuge tube with an open end. They were carefully released onto the feeder, where they began to imbibe sucrose solution. This process enabled the bees to associate the feeding station with the sucrose solution and establish a foraging route between the observation hive and the feeder. Typically, around 30 bees were trained before the experiment. When the trained bees had made approximately six trips to this feeder, we began to observe their waggle dances in the observation hive.

Per colony, we marked individual bees with unique combinations of colors and color dot locations on their thoraces using Edding 750 paint pens for identification. To regulate the number of bees on the dance floor, we opened the glass door covering the side of the colony with the dance floor and gently inserted a 35 cm × 17 cm metal wire fence with a mesh grid (grid spacing of 0.2 × 0.2 cm) into the comb around the dance floor (*SI Appendix*, Fig. S1). The 4 cm height of the fence was sufficient to prevent bees from climbing over it. Natural changes in the number of bees on the dance floor are mainly related to the return of bees from other foraging sites, but this is a slow, fluctuating process and does not drastically increase the number of bees on the dance floor. Therefore, the number of bees on the dance floor did not change significantly during the 10-min period of each audience-level phase (see below). This fence could have restricted the storage of collected nectar and the number of nectar unloaders. However, in this experiment, there was no effect of experimental phase on forager unloading delay time (*F*_3, 66_ = 1.30, *P* = 0.28, colony accounted for 8% of model variance and *R*^2^ = 0.13), suggesting that there was a sufficient number of workers to accept and potentially store incoming nectar during the times when the fence was in place. In the afternoon, approximately 1 h after the audience level was set, we recorded the waggle dance performances of marked bees using a high-definition video camera (HDR-PJ790, Sony Corporation). To increase image quality during video filming, we carefully opened the glass door of the observation hive and filmed the bees directly without the intervening glass.

To determine if variations in audience size altered the dance behavior of foraging bees, we manipulated the number of bees on the dance floor, with the glass door open. Each trial consisted of four 10-min intervals separated by 5-min intervals for the bees to adjust to these changes and had four different phases: High_control_ audience, Medium and Low audience (order pseudorandomly switched depending upon the trial), and High_recovery_ audience (*n* = 15 trials). The first phase was a control phase, and the number of bees on the dance floor was high (High_control phase_, mean ± 1 standard deviation, 251.3 ± 14.1 bees). In the next phase, we gently removed about half of the bees with an aspirator (DL881120, Ningbo Deli Tools Co., Ltd., *SI Appendix*, Fig. S5 and Movie S7) by haphazardly selecting bees at the dance floor’s edge to create the medium audience phase (Medium_phase_, 138.9 ± 20.0 bees) and recorded for another 10 min. Next, we removed approximately 75% of bees on the dance floor to create a low audience phase (Low_phase_, 59.1 ± 22.2 bees) and recorded for another 10 min. Alternatively, we created a Low_phase_ first and the Medium_phase_ next. We randomized the order of the Low_phase_ and Medium_phase_ after the High_control phase_. After the Low_phase_ or Medium_phase_, we removed the fence, allowing bees to return to the dance floor. After approximately 5 min, the number of bees on the dance floor restored itself to 92.8%, on average, of the original High_control phase_ level, and we therefore call this final phase High_recovery phase_ (mean ± 1 standard deviation, 233.1 ± 15.1 bees). During this High_recovery phase_ we recorded waggle dancing for another 10 min.

The aspiration technique that we used evidently did not disturb the remaining bees on the dance floor (see control experiments 1 to 3 below). Before we began aspiration, we briefly removed the feeder for about 5 to 6 min, causing the foragers to remain at the feeder site and search for the removed food. Thus, foragers did not immediately return to the nest and were not removed during the removal phase. Once the audience size manipulation was complete, we replaced the feeder, the foragers fed, returned to the nest, and our test phase began. At the end of the trial, the bees that had been removed by an aspirator (they were kept in the dark at 21 to 26 °C to maintain them in good condition) were released back into the colony. We measured the characteristics of the waggle dance for only one dance bout, defined as a bee entering the nest, beginning to dance, and then ending its dance to return and collect more food at the feeder (41). With each colony, we conducted five trials over five different days.

We counted the number of bees returning to the hive from the feeder within a 10-min phase and recorded the proportion of these returning bees performing a waggle dance, i.e., which we define as their propensity to dance, within this 10-min phase. To understand the relationship between dancing propensity and the number of bees on the dance floor during the 10-min phase, we counted and averaged the number of bees on the dance floor at the start and end of the 10-min phase, to measure the number of bees on the dance floor. We haphazardly selected marked foragers to measure the number of waggle runs per bee in one dance bout in response to the different audience sizes. At the beginning of the 10-min observation phase, we counted the total number of bees on the dance floor using a still image from the video. During the 10-min phase, we observed all dancers that appeared on the dance floor. The number of followers varies throughout a dance bout (19, 20), and thus, we calculated the average number of followers each dancer attracted during its dance bout. Following Rohrseitz and Tautz (27), we define a follower as a bee positioned up to one bee-width away from the dancer, facing the dancer, and following the dancer (its antennae track the dancer’s body) for an entire waggle run. We then summed these average follower counts across all dancers observed during the period to obtain the total number of dance followers on the dance floor.

### Measurements.

We measured the unloading delay time, the duration between a forager’s appearance on the dance floor and the start of its first unloading contact. Trophallaxis is defined as the exchange of liquid food between nestmates by regurgitation of the stomach contents of the food donor, and simultaneous imbibing by the receiver (42). Only trophallactic contacts ≥3 s were considered to be food unloading contacts (43). We counted the total number of trophallactic contacts beginning when a forager returned to the nest, while it danced, and after it danced and before it left the nest. We define each trophallactic contact as a bee touching its protruded proboscis to the prementum of the forager or the dancer touching its proboscis on the prementum of a worker (14). In experiment 1, the average trophallactic contact lasted for 4.2 ± 2.6 s (*n* = 72).

Because dancers produced fewer waggle runs per waggle dance in the Low _phase_, we selected bees with at least seven waggle runs per dance in this phase to meet the required sample size for the dance precision analyses, as recommended by Couvillon et al. (44). We used Tracker (v4.91) or Fiji ImageJ (v1.50i) software (see also control analysis 1, below), and the researchers making the measurements were blind to the treatment phase and the colony origin of the observed bee. We excluded the initial waggle run due to its variability and analyzed the subsequent six waggle runs (44).

For each dance, we measured: 1) the waggle run angle relative to gravity and then 2) calculated the standard deviation of waggle angles during six waggle runs (45) (Statistical Methods). We also measured 3) the duration of the waggle run (defined as the beginning and end of the dancer’s wing oscillations during a waggle run from the video filmed at 25 frames per s), 4) waggle duration variance (coefficient of variation of the waggle durations), 5) the number of waggles per waggle run (each waggle defined as one complete cycle from right to left to right movement of the abdominal tip), 6) variance in the number of waggles per waggle run (coefficient of variation of the number of waggles per waggle run per dance), and 7) the number of dance followers per waggle dance circuit (defined as one waggle run followed by one return run). We also calculated the average number of followers over six waggle runs to assess the impact of follower count on dance precision. For experiments 1 and 2, we counted the number of bees on the dance floor at the start of the dancer’s first waggle run to determine the waggle dance audience size.

### Quantifying Audience Seeking Behaviors.

We observed that waggle dancers appeared to exhibit “audience seeking” behavior when confronted with smaller audiences. Specifically, they tended to interrupt their waggle runs more frequently and moved around the dance floor, traveling greater distances and potentially encountering more bees compared to when they performed for larger audiences. To quantify this behavior, we compared the characteristics of the dancers’ first six waggle runs under conditions of high versus low audience size (High_control phase_ vs. Low_phase_). For each bee, we measured the total distance traveled starting from the point at which they began their first waggle run until they completed their sixth waggle run. We restricted our analysis to bees that performed at least seven waggle runs, as the final waggle run is typically more variable (44). This approach allowed us to determine whether initial waggle runs in low-audience phases were more divergent and less precise, potentially serving to attract additional followers. We hypothesized that dances performed before larger audiences would take less time and cover shorter distances to complete the same number of waggle runs. Conversely, dances performed under smaller audience conditions would likely exhibit increased “audience seeking” behavior, involving greater time spent and longer distances traveled to complete the same number of waggle runs.

### Experiment 2: Testing the Effect of the Number of Bees On the Dance Floor Vs. the Number of Followers.

Do dancers assess the number of bees on the dance floor or the number of actual followers? We designed experiment 2 to test whether the number of dance followers, bees that are actively seeking information from waggle dancers, not simply the number of bees on the dance floor, altered waggle dancing. We therefore manipulated the number of potential dance followers. Foraging and waggle dancing is controlled by age polyethism in honey bees, and bees less than 8 d old do not follow waggle dances (20). Thus, to regulate the number of dance followers, we controlled the number of young adult bees (<3 d old) on the dance floor.

Each trial consisted of two 10-min phases, and we conducted 15 trials with three colonies (*SI Appendix*, Table S2). During the first phase, the bees on the dance floor were all older adults that could follow waggle dancers (>14 d old, 103.7 ± 11.9 total bees). This first phase, therefore, had a high number of dance followers (High_adults_= 0 young bees). We recorded the waggle dance performances of marked foragers for 10 min. In the second phase, we gently removed most of the bees on the dance floor with an aspirator (see above), and then added a similar number of young bees to create a low number of dance followers (Low_adults_, 73.3 ± 6.8% young bees), but with a similar average number of total bees (106.8 ± 9.0 total bees, not statistically different from the High_adults_ phase, see Results). As in experiment 1, we removed the feeder before we began aspiration so that the trained foragers remained outside, searching for the removed food, and were therefore not aspirated. We then waited 5 min for the bees to acclimate, and we began to record waggle dancing and trophallaxis of foraging bees for 10 min.

In other trials, we created an intermediate level of young bees (Medium_adults_) by removing approximately 40% of adult bees (defined as bees >14 d old) on the dance floor and replacing them with a similar number of young bees (3 d old, 40.6 ± 4.9% young bees). We then repeated the same measurements as above. Between trials, we randomized the order of the Medium_adults_ and Low_adults_ phases after the initial High_adults_ phase by manipulating the number of young bees on the dance floor. With each colony, we conducted each trial five times over five different days.

We note that the fence could have restricted the storage of collected nectar and the number of nectar unloaders. However, there was no effect of experimental phase on forager unloading delay time (*F*_1, 57_ = 1.60, *P* = 0.21, colony effect <1%, *R*^2^ = 0.05), suggesting that there was a sufficient number of workers to accept and potentially store incoming nectar when the fence was in place.

#### Measurements.

In each phase, we calculated the proportion of marked bees performing the waggle dance after the foragers trained to the feeder returned to the dance floor over a 10-min period. We also counted the number of adult and young bees (defined as above) on the dance floor at the beginning and end of each 10-min period. To determine the effect of young vs. adult bees on forager bee dance precision and trophallaxis, we compared two different phases: “Medium adults” (young bees added to the dance floor, comprising 40.6 ± 4.9% of the dance floor population) and “High adults” (no young bees on the dance floor). Dance precision measurements for the waggle dance were the same as in experiment 1.

To measure the effects of trophallaxis on dancing, we analyzed the following parameters: the number of times that a foraging bee had trophallaxis with adult or young bees, and the total duration of this trophallaxis with adult bees or with young bees. We also measured the unloading delay time as described in experiment 1 (above). The mean trophallactic contact durations were 3.2 ± 2.0 s (*n* = 60) for adult bees and 0.1 ± 0.3 s (*n* = 59) for young bees.

We also measured potential “audience seeking” behaviors in this experiment, using the same methods as in experiment 1 (see above).

### Measuring the Effects of Potential Audience Sizes.

The number of followers attracted by a dancer can vary substantially, even when performed on dance floors with large potential audiences (Figs. 1 and 2). Some dancers manage to attract more followers even when there are few potential followers on the dance floor. Other dancers gain few followers, even on a dance floor with many potential followers. We hypothesized that dancers adjust their behavior based on their experiences of encountering either low or high potential audiences via quorum sensing immediately after they returned to the nest and during their first waggle run. Consequently, dancers would exhibit greater variability in their waggle dances under conditions of smaller potential audiences, even if the immediate number of followers remained constant. To test this hypothesis, we focused on dances attracting between two and five followers in experiments 1and 2, specifically comparing performances during the two phases exhibiting the largest differences in potential audience size. This method enabled us to directly compare dances with a similar number of followers (no significant differences in follower distributions, see Results) under different potential audience sizes, allowing us to examine the effect of potential audience size on dancing.

### Statistical Analyses.

All statistical analyses were performed using JMP Pro V16.1.0. We report mean ± 1 standard deviation. We conducted multiple independent, manipulative experiments using different sets of bees and colonies (*SI Appendix*, Table S1) to ensure robust replication. For data involving repeated measurements from individual bees (control experiments 1 and 4), we utilized repeated measures analyses (with bee identity as a random effect) to appropriately account for within-subject dependencies.

Our analyses were designed to test our specific hypotheses and were prespecified before data collection, closely following the framework established by Dong et al. (40), who demonstrated significant differences in dancing precision related to dancers’ ability to interact with experienced bees. Additionally, we conducted preliminary power analyses prior to initiating experiments. These power analyses guided our determination of appropriate sample sizes (*SI Appendix*, Table S1), to help ensure that our study possessed sufficient statistical power to detect biologically meaningful effects. We used Mixed Models (REML algorithm) with colony as a random effect and tested for all interactions, eliminating nonsignificant interactions and reporting the results of the simplified models.

Because prior studies have identified effects of food unloading wait time and trophallaxis on waggle dancing (12, 14, 16), we ran models for our main experiments (1 and 2) in which we tested the effect of unloading wait time, the number of trophallactic contacts, and the number of followers (all fixed effects) on dance errors (the coefficient of variation of waggle run durations and waggle angle standard deviations). Because waggle run angles are circular data (ranging from 0° to 360°), we quantified within-dance directional variability with the circular standard deviation, computed from the mean resultant length after converting angles to unit vectors using standard circular-statistics transformations (45). We did not use an angular coefficient of variation because the coefficient of variation relies on a linear mean and an interpretable ratio to that mean. The circular standard deviation is rotation-invariant and reflects angular concentration directly, so it provides a comparable measure of directional precision across dances without any additional scaling by the mean direction (45). In these models, colony was a random effect.

For experiment 2, when we examined the relationship between the average number of followers per waggle run and the number of waggle runs per performance, we log-transformed the number of waggle runs per performance based on residuals analysis.

We used Tukey HSD tests to make all pairwise comparisons corrected for potential Type I statistical error. Because we used different measures to analyze the effects of audience size (number of dance followers, number of bees on the dance floor, and audience phase sizes), we applied the Dunn-Sidak correction and denote significant values that pass this correction as ^DS^. For our analyses of potential audience seeking behavior, we used a different set of dancers from experiment 1 that were not included in any other analyses, and therefore these analyses did not require a Dunn-Sidak correction. To compare the number and duration of trophallactic contacts, we used paired two-tailed *t* tests.

For control experiment 4 (comb side experiment), we compared the same waggle dancers per colony on each side. We, therefore, ran repeated measures analyses with colony and bee identity as random effects.

To enhance the readability of our multiple control experiments and analyses, we detail the statistical methods for each of these experiments and analyses along with the results.

## Supplementary Material

Appendix 01 (PDF)

Movie S1. Waggle dance of a forager (orange and green paint on thorax) with a large mixed audience size (**experiment 1**).

Movie S2. Waggle dance of the same forager seen in **Video S1** (orange and green paint on thorax) with a small mixed audience size (**experiment 1**).

Movie S3. Waggle dance of a forager (orange paint on its thorax and right wing) in the presence of an audience primarily composed of young bees that do not follow waggle dancers. Bees with dark blue paint on their thoraces are young bees less than 3 days old.

Movie S4. Waggle dance of the same forager as **Video S3** (orange paint on its thorax and right wing) in the presence of a large adult audience size (**experiment 2**). On the left and top of the frames you can see the fence that we inserted into the dance floor (**Fig. S1**).

Movie S5. Trajectory of the same waggle dancer in the low audience phase.

Movie S6. Trajectory of a typical waggle dancer in the high audience phase.

Movie S7. The aspirator removes bees from the comb without disturbing the dancers performing the waggle dance.

## Data Availability

Data have been deposited in Zenodo (https://doi.org/10.5281/zenodo.10892587) (46). Study data are included in the article and/or supporting information.
