# Supplementary material for: The audience shapes the information content of the honey bee waggle dance
Source: Proc Natl Acad Sci U S A. 2026 Mar 23;123(14):e2518687123. doi: 10.1073/pnas.2518687123 (PMC13056074; doi:10.1073/pnas.2518687123)
Supplement: Supplementary file 1 — Appendix 01 (PDF) [file pnas.2518687123.sapp.pdf]

PNAS

Supporting Information for
The audience shapes the information content of the honey bee waggle dance

Tao Lin[1], Shihao Dong[1]*, Gaoying Gu[1, 2], Fu Zhang[1, 2], Xiuchuan Ye[1, 2], Tianyi Wang[1], Ziqi Wang[1, 2], Jianjun Li[1], James C. Nieh[3]*, Lars Chittka[4]*, Ken Tan[1]*

Shihao Dong
Email: dongshihao@xtbg.ac.cn

Ken Tan
Email: kentan@xtbg.ac.cn

Lars Chittka
Email: l.chittka@qmul.ac.uk

James C. Nieh
Email: jnieh@ucsd.edu

This PDF file includes:

Supporting text
Figures S1 to S8
Tables S1 to S2
Legends for Videos S1 to S7
SI References

SUPPLEMENTAL INFORMATION

DESCRIPTION OF CONTROL EXPERIMENTS & ANALYSES

Control experiments 1-3

We conducted these experiments to test the hypothesis that our aspiration technique disturbed dancers and that this disturbance, not the audience, led to our results. To facilitate clear interpretation, the methods and results for each experiment are presented together below.

Control analyses 1 & 2

We conducted these control analyses to test if our choice of software for measuring waggle dances could have contributed significant errors (**control analysis 1**) and if there was a systematic bias in the orientation of waggle angles that could have contributed to our results (**control analysis 2**). We also present these methods and results together (see below).

Control experiment 4

Finally, we conducted an independent replication of **experiment 1** to control for the potential disturbance of aspiration. In **experiment 1**, we waited *5 min* after aspiration before observing waggle dancers. In **control experiment 4**, we minimized potential disturbances by establishing different audience sizes on opposite sides of the colony the evening before testing, about *12 h*. We likewise present these methods and results together (see below).

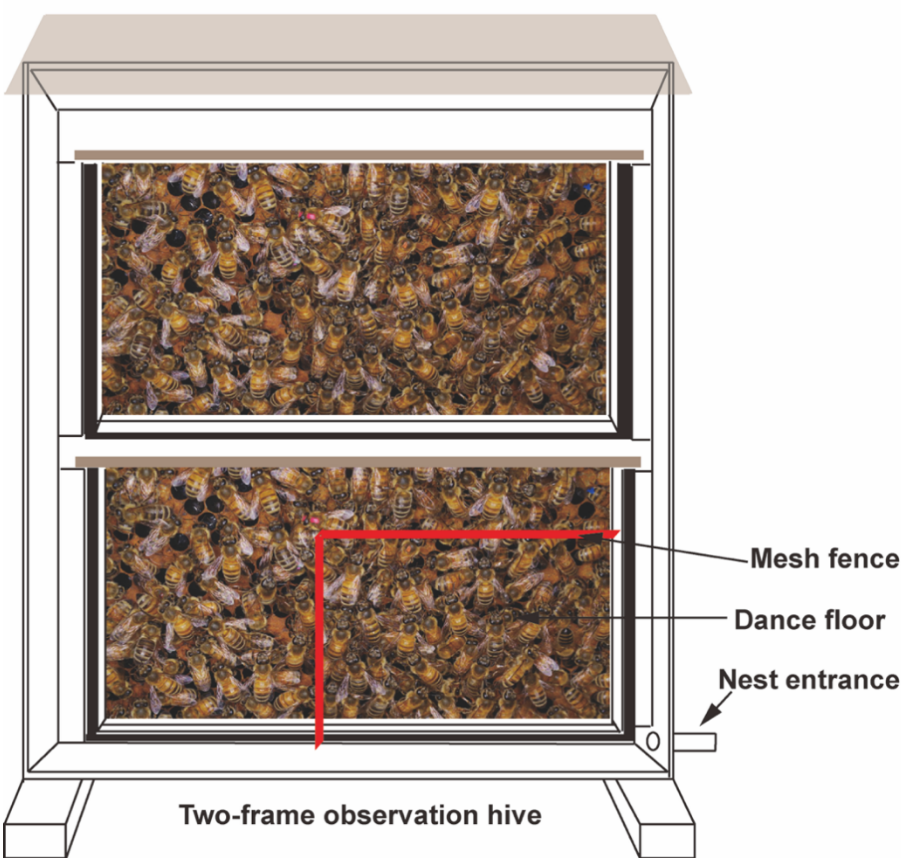

Figure S1. Schematic diagram of the bee observation hive. A mesh fence (red lines) was placed on the comb to restrict bees from other areas entering the dance floor. The placement of the fence ensured that returning foragers could only enter the area within the fence and, therefore, limited the number of bees on the dance floor. The fence did not affect forager unloading wait times.

Effects of phase and unloading delay time on trophallaxis

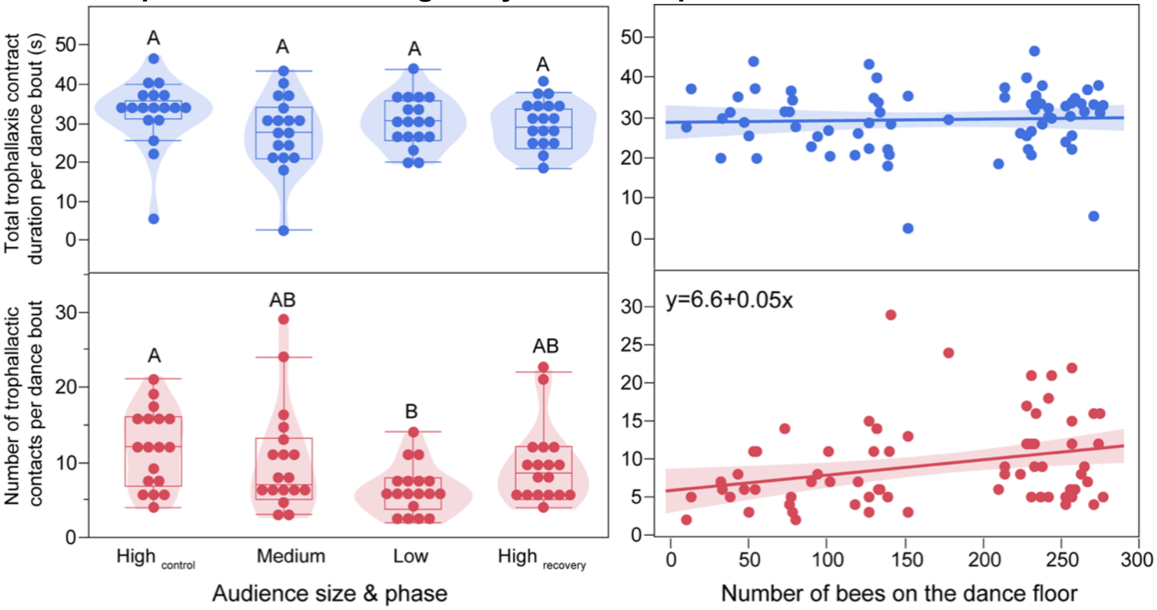

Figure S2. Forager trophallaxis decreased in the low audience phase (experiment 1). Low audience phase foragers had significantly fewer trophallactic contacts with nestmates than **High** $_{\textbf{control}}$ phase foragers ($F_{3, 66}$=3.49, P=0.02, colony accounted for 7% of model variance, R^2=0.19). For the number of trophallactic contacts, there were no significant effects of unloading delay time or the interaction of unloading delay time × number of bees on the dance floor ($F_{1, 67}$≤3.21, P≥0.08). There was no effect of audience phase on the total duration of trophallaxis per dance bout ($F_{3, 66}$=1.45, P=0.24, colony effect <1%). Different letters show significant differences (Tukey HSD test, P<0.05). There was a correlation between the total number of bees on the dance floor and the number of trophallactic contacts ($F_{1, 67}$=10.25, P=0.002 [DS1], colony accounted for 6% of model variance, R^2=0.19, the plotted line shows the fixed-effect fit from the mixed model with colony as a random effect but without unloading wait time as a factor). The number of bees on the dance floor did not predict the total duration of trophallactic contact ($F_{1, 68}$=0.10, P=0.76, colony accounted <1% of model variance).

In **experiment 2**, there were significantly fewer trophallactic contacts when the dance floor had a high proportion of young bees (in the low adults condition, 73% of dance floor bees were young bees, but only 4% of 1,149 contacts were with these young bees), and these contacts consisted only of 1% of the total trophallactic contact time (total contact time of 3,212 s, see Results for t-test results). On average, during 96 dance bouts, there were 11.5±5.0 contacts with adult bees and 0.4±0.8 contacts with young bees. Similarly, the mean duration of contact with adult bees was 33.1±10.1 s and 0.3±0.9 s with young bees (**Fig. S3**). For the total duration of trophallactic contacts with all bees, there were no effects of unloading delay time ($F_{1, 91}$=0.53, P=0.47), audience phase ($F_{1, 91}$=0.44, P=0.51), or their interaction ($F_{1, 91}$=0.89, P=0.34). For the total number of trophallactic contacts, there were no effects of unloading delay time ($F_{1, 93}$=0.65, P=0.42) or audience phase ($F_{1, 91}$=0.003, P=0.96), but there was a significant interaction of unloading delay time × audience phase ($F_{1, 91}$=5.88, P=0.02, 4% colony effect, R^2=0.10) because there was a significant increase in the number of trophallactic

[1] DS = Dunn–Šidák correction

contacts with unloading delay time in the **High** $_{\textbf{adults}}$ phase, when there were no young bees on the dance floor, but not in the **Medium** $_{\textbf{adults}}$ phase.

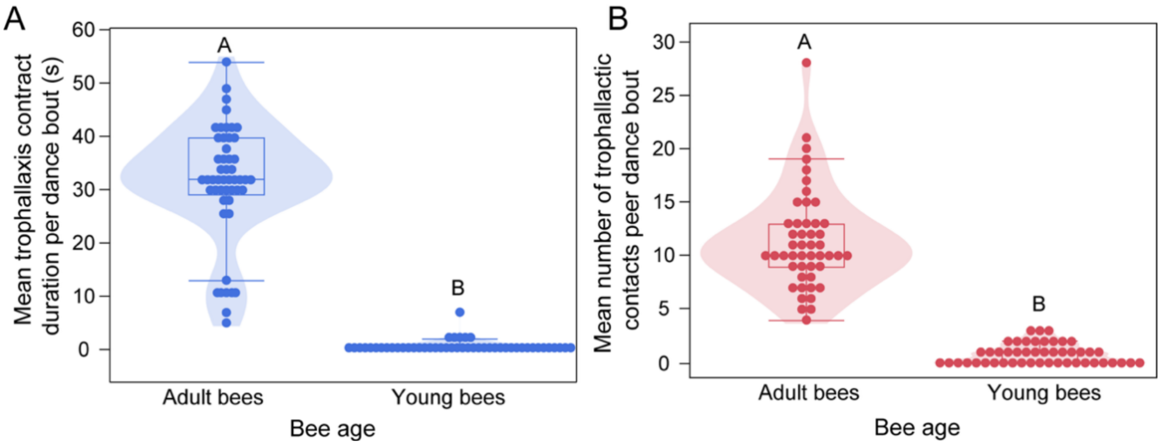

Figure S3. **An audience composed largely of young bees, which ignore dancers, also reduced forager trophallaxis (experiment 2).** (A) Foragers spent more time engaging in trophallaxis (P<0.0001) and (B) had more trophallactic contacts with adult bees than with young bees. Different letters show significant differences (P<0.0001).

There was no relationship between the number of dance followers and trophallaxis

An interesting question is whether the number of dance followers is associated with the total number and duration of trophallactic events experienced by a forager when it enters the nest to unload its collected nectar, dances, and then before it returns to collect more nectar. We therefore ran a Mixed Model (REML algorithm, see above) with the number of dance followers as a fixed effect and colony identity as a random effect.

In **experiment 1**, there was no significant effect of the number of followers on the *total number* of trophallactic contacts ($F_{1, 68}$=4.49, P=0.04 $^{\text{not significant after DS correction}}$, colony effect=7%, R^2=0.12) or on the *total duration* of trophallactic contacts ($F_{1, 69}$=0.72, P=0.40, colony effect <1%, R^2=0.25). Likewise, in **experiment 2**, there was no significant effect of the number of followers on the *number* of trophallactic contacts ($F_{1, 38}$=0.46, P=0.50, colony effect <1%, R^2=0.01) or on the *total duration* of trophallactic contacts ($F_{1, 58}$=0.95, P=0.33, colony effect <1%, R^2=0.10). These results are consistent with prior work indicating that most trophallactic events occur before and after dancing (1, 2) and therefore may not be correlated to the number of dance followers. Our results show that trophallaxis between a waggle dancer and its followers does not significantly predict the overall number or duration of trophallactic contacts that this nectar forager receives when it returns to the nest.

Name	Definition	Rationale
**Experiment 1**: Altering the total audience size	We manipulated the total number of bees on the dance floor (except for the number of marked nectar foragers, which were fixed). Bees came from the same genetic background as the source colonies and were all ≥14 days of age.	How the number of bees on the dance floor affects dance motivation and dance precision in foraging bees.
High $_{\text{control phase}}$	We did not remove any bees to manipulate the number of bees on the dance floor. The number of bees on the dance floor was 251.3±14.1 bees (mean±1 standard deviation).	Control phase
Medium $_{\text{phase}}$	We removed approximately half of the bees on the dance floor. The bees on the dance floor were 138.9±20.0 bees, 55.3% of the original bee population.	When the number of bees on the dance floor is halved, how does this affect dancing?
Low $_{\text{phase}}$	We reduced the audience size on the dance floor to 23.5% of its original size (59.1±22.2 bees).	When the number of bees on the dance floor is reduced to about one quarter of the original, how will this affect dancing?
High $_{\text{recovery phase}}$	We removed the fence and allowed bees to return to the dance floor. The bee population then returned to 92.8% of its original size (233.1±15.1 bees).	Can we restore original dance behavior by restoring the original audience size?
**Experiment 2:** Constant number of bees, but changing the number of potential followers	The audience on the dance floor consisted of a mixture of young and adult bees. The young bees were under 3 days old ***and did not follow dances***. The adults were 14 days old or older and followed dances.	Does the number of bees on the dance floor matter or are dancers paying attention to the number of bees that follow their dances?
High $_{\text{adults}}$	The dance floor consisted of 100% adult bees that were ≥14 days old (103.7±11.9 bees).	Control phase
Medium $_{\text{adults}}$	The dance floor consisted of a mixture of young and adult bees, of which young bees accounted for 40.6±4.9% of the bees (100.5±11.3 total bees).	What happens to waggle dancing when the proportion of adult bees decreases?
Low $_{\text{adults}}$	The dance floor contained a high proportion of young bees, who comprised 73.3±6.8% of the bees (106.8±9.0 total bees).	What happens to waggle dancing, when there are very few adult bees that can become followers?

Table S1. Definitions of the different phases and the rationales for the main experiments.

Experiment	Description	Colonies used	Number of different bees whose waggle dances were measured in this experiment or trials
1	Audience size (altering total audience)	A	24 bees
		B	24 bees
		C	24 bees
2	Young bee experiment	D	20 bees
		E	20 bees
		F	20 bees
Ctrl 1	Comparing dances before and after aspirator removal of followers	C1	5 bees
		C2	5 bees
		C3	5 bees
Ctrl 2	Comparing effects of aspiration vs. forceps removal	C1	6 bees
		C2	6 bees
		C3	6 bees
Ctrl 3	Measuring alarm volatiles (SPME) resulting from aspiration vs. forceps removal	C1	9 trials
		C2	9 trials
		C3	9 trials
Ctrl 4	Different audiences in the same colony (side A vs. side B)	H	12 bees
		I	12 bees
		J	12 bees
Ctrl Analysis 1	Comparing ImageJ with Tracker analysis precision	C4	5 bees
		C5	5 bees
		C6	7 bees
Ctrl Analysis 2	Investigating the potential for directional error due to gravity orientation of waggle angles	A	19 bees
		B	13 bees
		C	17 bees

Table S2. Experimental sample sizes

CONTROL EXPERIMENTS & CONTROL ANALYSES

We tested for possible disturbance effects of bee removal by aspirators on waggle dancing (**three control experiments**, **SI**), but found no evidence for significant disturbance effects or that such potential effects contributed to our results (**Figs. S4-S6**).

Control experiment 1: Testing if removing bees with aspirators disturbs waggle dancing

To test if aspiration disturbed waggle dancing, we haphazardly selected waggle dancers trained to a 60% w/v sucrose solution located 500 m from the focal colony. After videotaping the waggle dance for six waggle runs, we used an aspirator to remove bees near the dancer (within 10 cm) for about 10 s. Then, we continued to video record another six waggle runs of that dancer. We repeated this for five bees per colony with three colonies and measured waggle angle standard deviation, waggle run duration, coefficient of variation of waggle run duration, the number of waggles per waggle run, and the coefficient of variation of number of waggles per waggle run using the same software and methods as described for **experiment 1**. For this experiment, we ran univariate repeated measures models with bee as the repeated measure since we measured the same waggle dancer before and after a bee was aspirated next to the dancer.

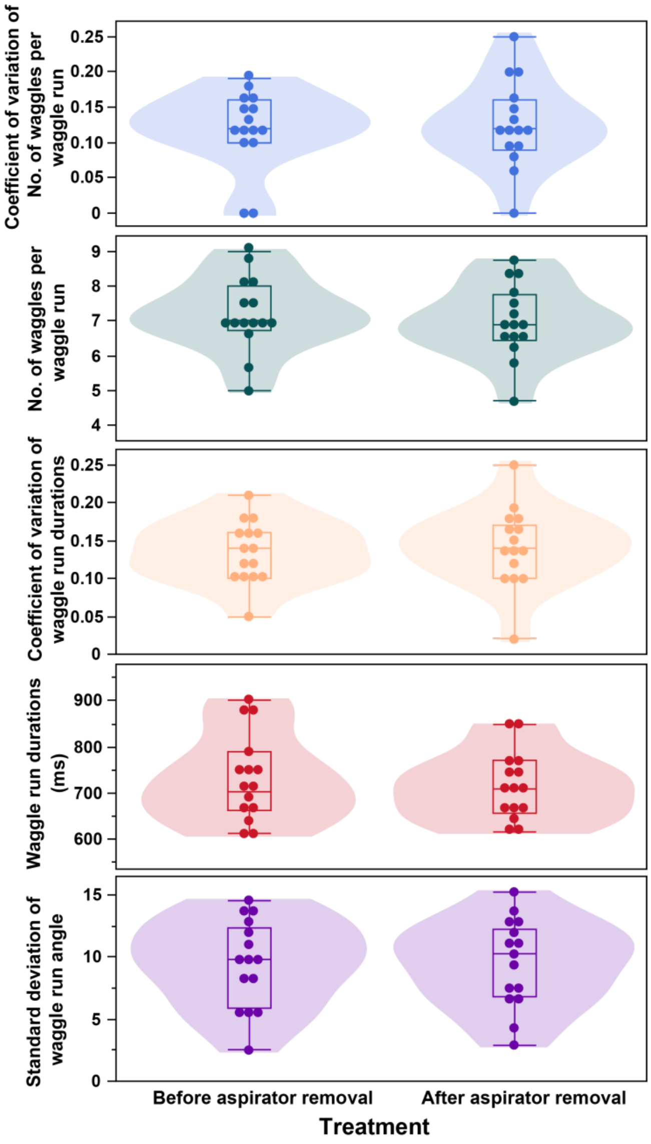

Figure S4. There were no significant effects of aspirator removal on nearby waggle dancing bees whose dances were recorded immediately before and after an adjacent bee was aspirated ($P \geq 0.60$).

Results: Waggle dancers were not disrupted after an adjacent non-follower was removed with an aspirator

There was a delay of 5 min between each phase. Potentially, the disturbance effect would not be detected after 5 min. We therefore looked for an immediate disturbance effect. However, aspirating a bee next to a waggle dancer did not affect waggle dancing (**Video S7**). We measured the same waggle dances immediately before and after aspiration and found no significant differences in waggle angle standard

deviation, waggle run durations, coefficient of variation of waggle run durations, the number of waggles per waggle run, or coefficient of variation of the number of waggles per waggle run ($F_{1, 22}$≤0.59, P≥0.45, **Fig. S4**).

Control experiment 2: Disturbance effects of aspiration vs. removal with forceps

To determine if aspiration potentially disturbed bees, we compared removing bees with the aspirator or with forceps whose metal tips were covered with silicone tubing (**Fig. S5**). Previously, Seeley manipulated the number of food-storing bees in observation colonies by removing them with padded forceps (3). Seeley observed that food-storing bees are older than nurse bees and significantly younger than foragers and primarily focused on receiving and processing nectar (3). Although food-storers could become dance followers, such following was mainly observed in hives nearly full of honey, unlike our colonies, which had ample space to store additional honey. Thus, the removal of food-storing bees in his groundbreaking experiment altered the waggle dancing audience, to some degree, but was not focused on testing the hypotheses that we tested.

To compare the effects of aspirator versus forceps removal, we removed bees with both methods for 1 min each. We counted the number of removed bees, as well as the instances of fanning, lunging, and aggression (the bee grabbing the tested device with its legs or mandible and trying to sting it) towards the removal device. For our statistical analyses, we used Mixed Models (REML algorithm) with colony as a random effect and removal method as a fixed effect.

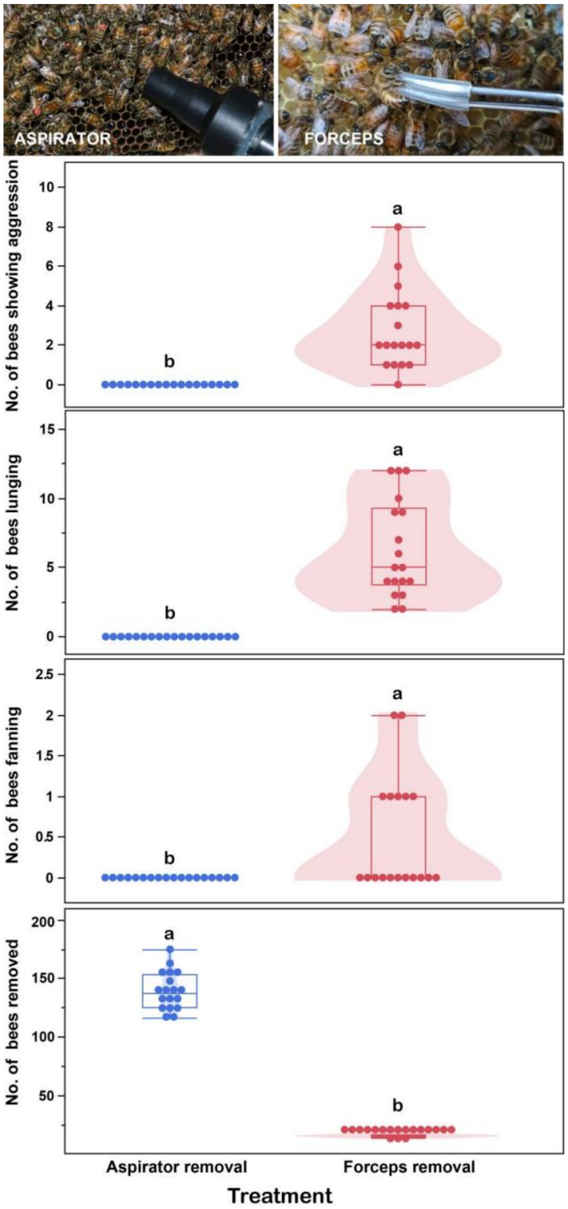

Figure S5. Forceps removal of a bee resulted in significantly more disturbance to an adjacent bee than aspirator removal of a bee (P<0.0037) although more bees were removed per min by the aspirator than by the forceps. Aggression is defined as the bee grabbing the tested device with its legs or mandible and trying to sting it. Different letters indicate significant differences (Tukey HSD test, P<0.05).

Results: Forceps removal was disturbing, but aspirator removal was not

We next compared our aspiration treatment with a standard method of removing bees from the comb in behavioral experiments: removal with forceps. Forceps removal was significantly more disruptive than aspirator removal within 1 min by all tested measures ($F_{1, 32}$=9.82, P≤0.0037). This result is also striking because the aspirator removed 8.5-fold more bees per min than the forceps. The aspirator removed 139.3±3.6 bees per min, and the forceps removed 16.3±0.4 bees per min (mean±1 standard error). If the aspirator was disturbing bees, bystanders should be fanning, lunging, or showing highly aggressive behavior towards the aspirator. However, aspirator removal never resulted in neighboring bees fanning or lunging or showing highly aggressive behavior towards the aspirator (**Fig. S5**). Thus, although the disturbance caused by forceps removal likely depends upon the skill of the remover, even with careful removal, we found it significantly more disruptive than aspirator removal.

Control experiment 3: Detection of potential alarm pheromone volatiles with SPME

To detect potential alarm pheromone volatiles produced by bees removed with an aspirator or forceps, compared to a control group of undisturbed bees, we employed solid-phase microextraction (SPME). We applied one of three treatments: aspirator removal, forceps removal, or control. For the aspirator treatment, we positioned a clean PTFE tube (1.5 mm diameter) approximately 1 cm from the center of the dance floor without direct contact. Inside the PTFE tube, we placed a 65-μm PDMS/DVB SPME fiber (Supelco). We sampled air using a pump with a flow rate of 1 mL/s. Initially, we started the air pump and then used an aspirator to remove bees near the tube for 1 min. After stopping the aspiration, we continued to draw in air with the pump for an additional 29 min. For the forceps treatment, we also used a pump to draw air from a clean PTFE tube placed approximately 1 cm from the center of the dance floor. When the pump was started, the bees moved towards the center of the dance floor. We used forceps to pinch and remove bees near the tube, placing them in a bottle away from the nest. This pinching operation was repeated for 1 min, removing about 16 bees. After stopping the pinching, we continued to draw air with the pump for another 29 minutes. For the control treatment, we positioned the PTFE tube approximately 1 cm from the center of the dance floor without direct contact with any bees and sampled air for 30 min (1 min + 29 min). As the standards, we used SPME fibers to collect one bee-equivalent of two major volatile honey bee sting alarm pheromone components, 3-methyl-1-butanol (MB) and isopentyl acetate (IPA), in a clean glass vial for 10 s, followed by GC-FID analysis.

For chemical analysis, we used a gas chromatography-flame ionization detector (GC-FID) with an HP 7890B gas chromatograph (Agilent, Santa Clara, CA, USA) and an HP-5 column (30 m × 0.32 mm × 0.25 μm, Agilent). We used helium as the carrier gas at a flow rate of 37 cm/s. The oven temperature was programmed to hold at 50°C for 2 minutes, then increase by 10°C per minute to 280°C for 10 min. Each SPME fiber was desorbed into the injector port at 250°C for 1 minute. We replicated each treatment three times per colony. We then measured the areas under the curves for two abundant honey bee alarm pheromone components: isopentyl acetate (IPA) and 3-methyl-1-butanol (MB) (4).

For our statistical analyses, we used Mixed Models (REML algorithm) with colony as a random effect and treatment as a fixed effect.

Results: Removal by aspirators did not result in the detectable release of alarm pheromone volatiles

Did removing bees with aspirators release sting alarm pheromone? In our measurements, only pinched bees produced the major alarm pheromone volatiles (3-methyl-1-butanol, **MB**; isopentyl acetate, **IPA**). We never detected any MB or IPA from aspirated or control bees ($F_{2, 47}$=83.86, P<0.0001, colony effect =15%, **Fig. S6**). The non-detection of alarm pheromone compounds is not sufficient to demonstrate that no alarm pheromones were produced, but given the results detailed above, we found no evidence supporting this hypothesis, unlike forceps removal, which was clearly disruptive.

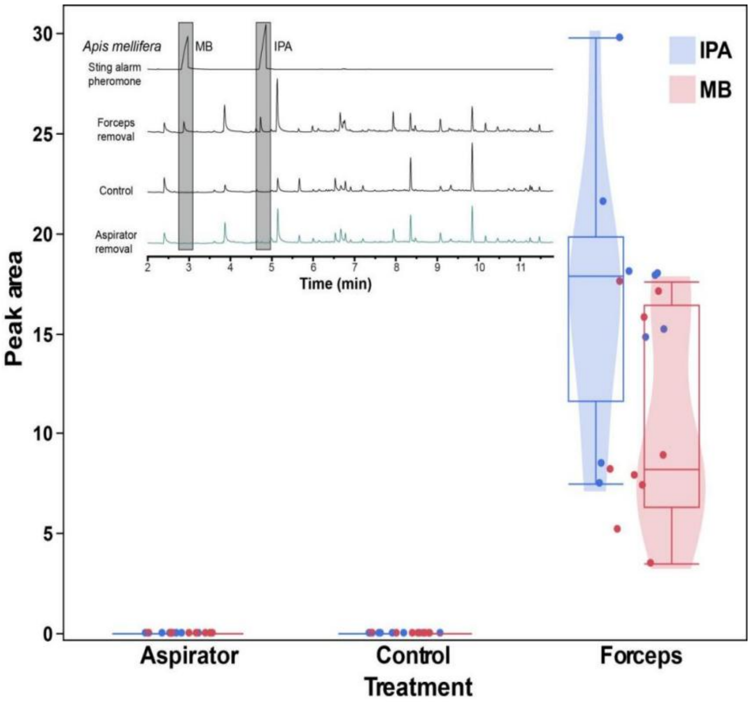

Figure S6. No detectable production of major alarm pheromone volatiles around bees as a result of aspirator removal. The inset shows typical chromatograms revealing the volatiles from *Apis mellifera* sting alarm pheromone (see methods). We focused on two highly volatile components of honey bee sting alarm pheromone: 3-methyl-1-butanol (**MB**) and isopentyl acetate (**IPA**). Forceps removal resulted in significantly higher production of MB and IPA, which we never detected from the aspirator removal (different letters show significant differences, Tukey HSD test, P<0.05).

Summary of the control experiments testing the aspiration disturbance hypothesis

Thus, the hypothesis that aspirating the bees disturbed their dancing and therefore accounts for our results was not supported by 1) testing the immediate effects of aspiration on waggle dancing, 2) comparing aspiration with forceps removal, or 3) testing for the presence of alarm pheromone. It remains possible that aspiration did disturb dancing bees or other bees on the dance floor, but we found no evidence of such disruptive effects in these multiple measures of potential disturbance.

Control experiment 4: Minimizing potential disturbances from manipulating audience size (high vs. low audience on different sides of the same comb)

Although our multiple control experiments (**control experiments 1-3**) did not detect any disturbance effects resulting from the aspiration used to manage audience size, *we conducted a second, independent experiment with new colonies in a different field season to minimize potential disturbances by establishing different audience sizes on opposite sides of the colony about 12 h before testing*. Our goal was to reduce any potential effects of bee removals on the dancers by allowing more time between the manipulations and waggle dancing and testing each forager on both sides. In this

experiment, we used three colonies, each containing approximately 1,200 adult bees over 14 days old. As in our earlier study, we trained the bees to visit a feeder, and once they did so reliably, they began performing waggle dances. We conducted this experiment from August to October in 2024.

We set up the observation hive so that one side of the comb (side A, high audience) housed significantly more bees than the other side (side B, low audience). On the evening before data collection, after most foragers had returned to the hive, we gently transferred half of the bees from the low-audience side to the high-audience side using a standard bee brush. We sealed the edges of the comb with beeswax to prevent bees from traveling from one side to the other. Bees could continue to exit and enter the colony, but very few did so, given that it was evening and bees typically do not leave the colony or return at night.

In the morning, about 12 hours later, bees departed to begin foraging, and when our trained and marked bees returned to the hive from the feeder and began dancing, we randomly directed them to either side by adjusting a small wooden barrier at the hive entrance. When a forager left the feeder, the feeder monitor notified the nest observer with a two-way radio, and the nest observer was then able to direct the forager to either the high-audience or the low-audience side by moving a wooden slider before the bee forager entered the nest, thereby minimizing any potential disturbance. For example, if bee 1 first danced on the high-audience side, the next time it returned to the nest, we shifted the wooden divider to guide it to

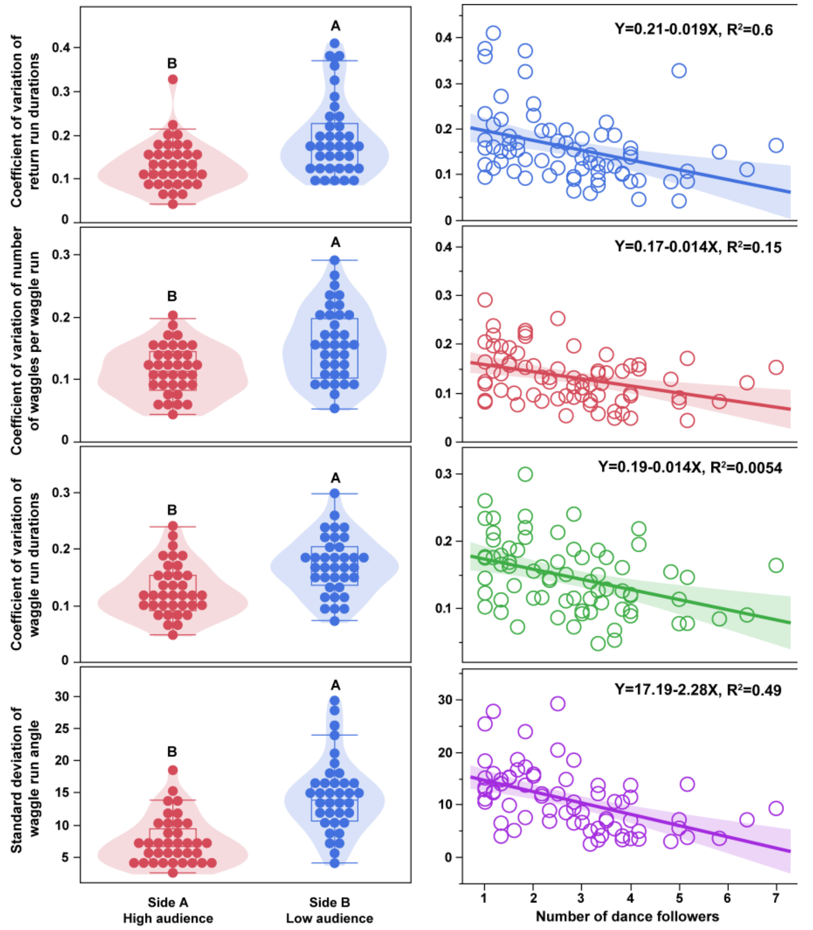

Figure S7. When the number of bees present around the dance area was manipulated on opposite sides of the same comb, waggle dances consistently showed lower precision in the presence of a smaller (low audience) group. On the high audience side, bees exhibited lower standard deviation in waggle dance angles, as well as lower coefficients of variation in waggle duration, the number of waggles per waggle run, and return run duration—indicating greater dance precision compared to the low audience side. Different letters show significant differences (Tukey HSD test, *P*<0.05).

dance on the low-audience side. Half of the foragers were directed to begin on side A, while the other half were initially sent to side B.

Each forager danced on both sides, with an interval of approximately 5 min between dances. Dancer motivation can shift with time, and so we chose to test each dancer within a short period of time to minimize motivational shifts. We recorded all waggle dances using a high-definition video camera (HDR-PJ790, Sony Corporation), following the same procedures used in our previous experiments.

Results: Minimizing potential disturbances from manipulating audience size (high vs. low audience on different sides of the same comb) yielded the same results as experiments 1 & 2.

On average, the high-audience side had 864.4±27.4 bees (3.0±1.3 dance followers) while the low-audience side had 372.9±14.7 bees (1.6±0.7 dance followers, significantly fewer: $F_{1, 68}$=134.91, P<0.0001).

Consistent with our other experiments, foragers had significantly lower waggle angle standard deviations on the high-audience side than when the same foragers danced on the low-audience side ($F_{1, 35}$=43.35, P<0.0001 ^{DS}, **Fig. S7**). There were no significant differences in waggle run durations ($F_{1, 35}$=0.46, P=0.50), the number of waggles per waggle run ($F_{1, 35}$=0.43, P=0.52), or return run durations ($F_{1, 35}$=1.19, P=0.28, **Fig. S7**). However, there was significantly greater variation in these measures: coefficient of variation of waggle run durations ($F_{1, 35}$=15.61, P=0.0004 ^{DS}), coefficient of variation of number of waggles per waggle run ($F_{1, 35}$=11.86, P=0.0015 ^{DS}), and coefficient of variation of return run durations ($F_{1, 35}$=12.07, P=0.0014 ^{DS}, **Fig. S7**). *Thus, dancers communicated more variable distances and food quality when they had smaller audiences.*

In addition, dancers also had significantly lower waggle angle standard deviations when they had more *followers* compared to when the same dancers had fewer followers ($F_{1, 44}$=29.16, P<0.0001 ^{DS}). There were no significant differences in waggle run durations ($F_{1, 42}$=0.44, P=0.51), the number of waggles per waggle run ($F_{1, 43}$=0.21, P=0.65), or return run durations ($F_{1, 42}$=1.14, P=0.29). However, we observed significantly greater variability in these measures on the low-audience side: coefficient of variation of waggle run durations ($F_{1, 52}$=10.79, P=0.0018 ^{DS}), coefficient of variation of number of waggles per waggle run ($F_{1, 55}$=9.89, P=0.003 ^{DS}), and coefficient of variation of return run durations ($F_{1, 60}$=8.37, P=0.005 ^{DS}). *Thus, when given smaller audiences, dancers communicated more variable distances and food quality even when potential disturbance effects from creating different audience sizes were minimized and when temporal effects were also minimized* (the same dancer was tested within approximately 5 min on each colony side).

Control analysis 1: Checking the agreement of Tracker vs. ImageJ software

Tracker video analysis software (Open Source Physics Project) is widely used, but may be less accurate compared to ImageJ, a commonly utilized image analysis software. We conducted our image analyses using Tracker software (Tracker 4.91), as described in **experiment 1**. To determine if similar results could be obtained using another standard image analysis package, Fiji ImageJ v1.50i (5), we measured the same videos of waggle dancing using both software packages. We then used a standard test of reliability, calculating the linear correlation between data collected with Tracker with data collected on the same video with Fiji ImageJ. We analyzed at least five videos

per colony (each of a different waggle dancer) from three different colonies and measured waggle angle standard deviations, waggle run durations, the number of waggles per waggle run, and the return run durations. We plotted these data (**Fig. S8**) and calculated the linear regression equations and coefficients.

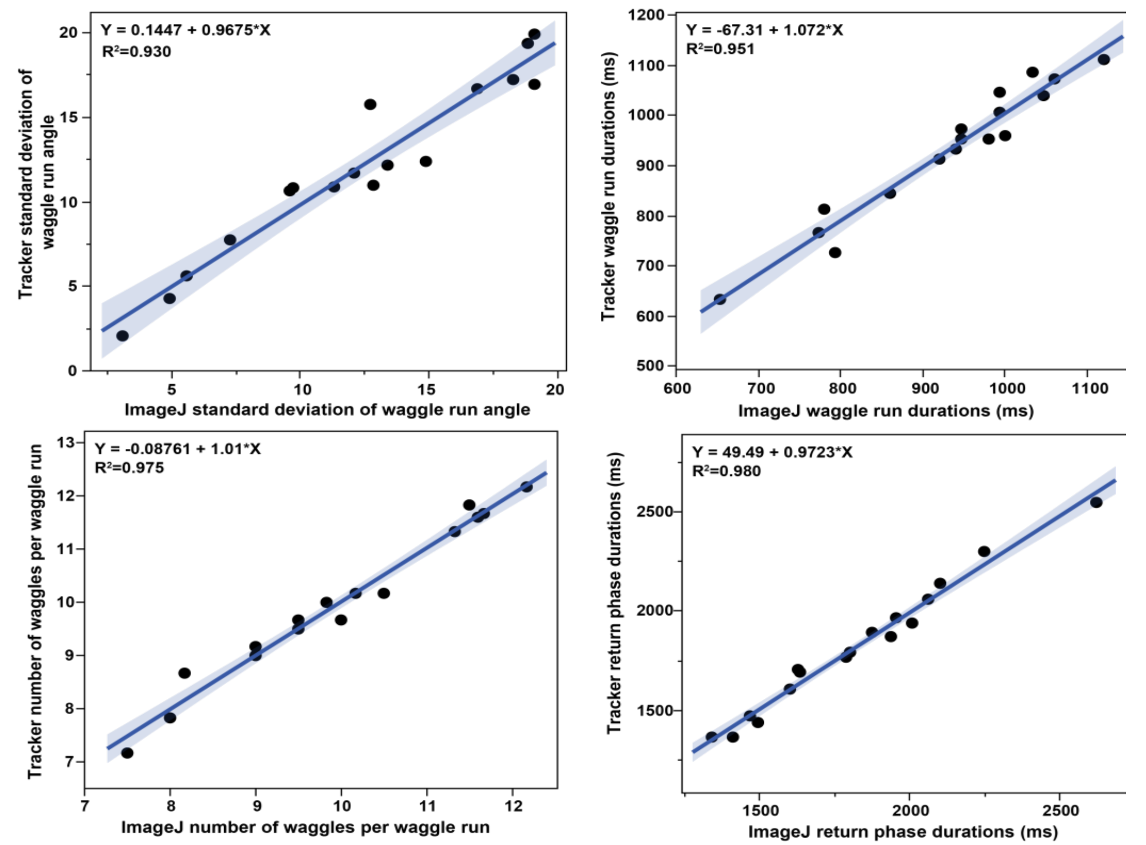

Figure S8. **Agreement between waggle dance measurements made with Tracker vs. Fiji ImageJ software.** Linear regression lines, equations, and R^2 values are shown (17 videos from three different colonies analyzed, with each video corresponding to a different bee). Shaded areas show the 95% confidence intervals.

Results: Tracker and ImageJ software analyses were in good agreement

There was strong agreement between the video data collected from the same video using Tracker v4.91 as compared to ImageJ software v1.50i (National Institutes of Health, USA) (**Fig. S8**). The regression line slopes ranged from 0.97 to 1.0 and the linear correlation coefficients ranged from 0.93 to 0.98. Thus, the Tracker software was adequate for our measurements.

Control analysis 2: Waggle angle directions did not account for the observed errors

Couvillon et al. demonstrated that honey bee waggle dances performed at more horizontal orientations exhibit significantly greater angular scatter than dances oriented near the vertical, indicating that gravitational forces contribute to directional variability (6). Following their approach, we expressed waggle-run orientations on a 0°–180° scale relative to vertical. To avoid artifacts caused by the circular boundary at 0°/360° when a

within-dance set of waggle-run angles straddled that boundary, we applied a wrap-around correction by adding 360° to the smaller angles (for example, 10° → 370° when paired with 350°) and then applying an equal constant rotation to all angles in that dance to obtain one continuous cluster within a 180° window. Because this procedure only adds or subtracts a constant within a dance, it preserves within-dance angular separations, and linear standard deviations are appropriate once the angles are continuous. Inspection of the phase-specific angle distributions showed unimodal, approximately symmetric distributions, and we therefore fit Mixed Models (REML algorithm) with audience size phase as a fixed effect and colony as a random effect.

In experiment 1, we varied the number of bees on the dance floor, yielding mean angles of 103°, 101°, 103°, and 103° for the High control, High recovery, Low, and Medium phases, respectively. There was no significant effect of audience size phase ($F_{3,66}$=0.21, P=0.89), and colony accounted for 2% of model variance.

In experiment 2, we kept the total number of bees on the dance floor fairly constant but reduced the number of dance followers by increasing the proportion of young bees, which do not follow dances. Under these conditions, the mean waggle angles were 30±15° (High adults, more followers) and 42±10° (Medium adults, fewer followers). We found a significant effect of phase ($F_{1,\ 56}$=12.85, P=0.0007), with colony accounting for 3% of the model variance. However, using the regression equation reported by Couvillon et al. shows that shifting the mean waggle angle from 30° to 42° should increase the predicted angular standard deviation by only about 0.47° (6). This predicted gravity-related change is much smaller than the observed 5° difference in waggle-angle standard deviations between phases, indicating that the increased directional error cannot be explained by the modest shift in mean dance orientation and is instead associated with reduced follower availability.

VIDEO EXAMPLES

Video S1. Waggle dance of a forager (orange and green paint on thorax) with a large mixed audience size (**experiment 1**).

Video S2. Waggle dance of the same forager seen in **Video S1** (orange and green paint on thorax) with a small mixed audience size (**experiment 1**).

Video S3. Waggle dance of a forager (orange paint on its thorax and right wing) in the presence of an audience primarily composed of young bees that do not follow waggle dancers. Bees with dark blue paint on their thoraces are young bees less than 3 days old.

Video S4. Waggle dance of the same forager as **Video S3** (orange paint on its thorax and right wing) in the presence of a large adult audience size (**experiment 2**). On the left and top of the frames you can see the fence that we inserted into the dance floor (**Fig. S1**).

Video S5. Trajectory of the same waggle dancer in the low audience phase.

Video S6. Trajectory of a typical waggle dancer in the high audience phase.

Video S7. The aspirator removes bees from the comb without disturbing the dancers performing the waggle dance.

REFERENCES

1. R. J. De Marco, How bees tune their dancing according to their colony's nectar influx: re-examining the role of the food-receivers' 'eagerness'. *J. Exp. Biol.* **209**, 421-432 (2006).
2. R. J. De Marco, W. M. Farina, Trophallaxis in forager honeybees (*Apis mellifera*): resource uncertainty enhances begging contacts? *J. Comp. Physiol. A* **189**, 125-134 (2003).
3. T. D. Seeley, Social foraging in honey bees: how nectar foragers assess their colony's nutritional status. *Behav. Ecol. Sociobiol.* **24**, 181-199 (1989).
4. B. R. Wager, M. D. Breed, Does honey bee sting alarm pheromone give orientation information to defensive bees? *Ann. Entomol. Soc. Am.* **93**, 1329-1332 (2000).
5. R. Schürch *et al.*, Dismantling Babel: creation of a universal calibration for honey bee waggle dance decoding. *Anim. Behav.* **150**, 139-145 (2019).
6. M. J. Couvillon, H. L. F. Phillipps, R. Schürch, F. L. W. Ratnieks, Working against gravity: horizontal honeybee waggle runs have greater angular scatter than vertical waggle runs. *Biol. Lett.* **8**, 540-543 (2012).
